# *Ab Initio* Insights into the Interfacial Interactions of Emerging Two-Dimensional Nanomaterials with C‑A-S‑H Geopolymer Matrices

**DOI:** 10.1021/acsomega.6c04834

**Published:** 2026-10-06

**Authors:** Rabindranath Lo, Anurag Misra, Kolleboyina Jayaramulu

**Affiliations:** † Institute of Organic Chemistry and Biochemistry, 89220 Czech Academy of Sciences, v.v.i., Flemingovo nám. 2, Prague 6 16000, Czech Republic; ‡ Department of Civil Engineering, 528895 Indian Institute of Technology Jammu, Jammu and Kashmir 181221, India; § Hybrid Porous Materials Laboratory, Department of Chemistry, 528895 Indian Institute of Technology Jammu, Jammu and Kashmir 181221, India; # Department of Civil Engineering, National Institute of Technology Srinagar, Hazratbal, Srinagar, Jammu and Kashmir 190006, India

## Abstract

The development of sustainable construction materials has positioned geopolymer concrete as a promising alternative to conventional Portland cement due to its reduced carbon footprint and utilization of industrial byproducts. In this context, two-dimensional (2D) materials have emerged as versatile reinforcing fillers that enable the precise tuning of interfacial interactions, thereby improving the mechanical and physicochemical performance of geopolymer systems. Herein, we present a systematic first-principles investigation of a diverse class of two-dimensional (2D) materials, including Xenes (silicene, germanene, stanene, arsenene, tellurene, and blue phosphorus), transition metal dichalcogenides (MoS_2_ and WS_2_), MXenes, graphene derivatives (including graphdiyne), and ceramic/covalent systems such as SiC, boron phosphate, boron nitride, and CrO_2_ by examining their interfacial interactions with a structurally ordered calcium aluminosilicate hydrate (C-A-S-H) model using density functional theory (DFT). DFT-guided calculations reveal a clear hierarchy in interfacial binding, ranging from strong chemisorption (≈−1.5 to −4.0 eV) for MXenes and functionalized graphene derivatives, to moderate interactions (≈−0.8 to −1.5 eV) for Xenes and carbides, and weak physisorption (≈−0.2 to −0.8 eV) for transition metal dichalcogenides (TMDs) and ceramic surfaces. MXenes and functionalized graphene exhibit the strongest adhesion, driven by covalent bonding, Ca^2+^ mediated bridging, and charge transfer, leading to improved load transfer and microstructural densification. In contrast, graphdiyne and h-BN are dominated by van der Waals interactions, contributing mainly to durability and stability. Overall, this study establishes that surface functionality and electronic structure govern interfacial strength and reinforcement efficiency in 2D material-geopolymer systems. Therefore, this DFT-guided comparative study establishes a unified structure–interaction–property relationship and highlights that surface functionality and electronic structure are the key descriptors governing reinforcement efficiency. The findings establish clear design principles for the rational selection of 2D reinforcements, positioning MXenes, Xenes, and functionalized graphene as leading candidates for the development of next-generation, high-performance, and durable geopolymer concretes.

## Graphical abstract

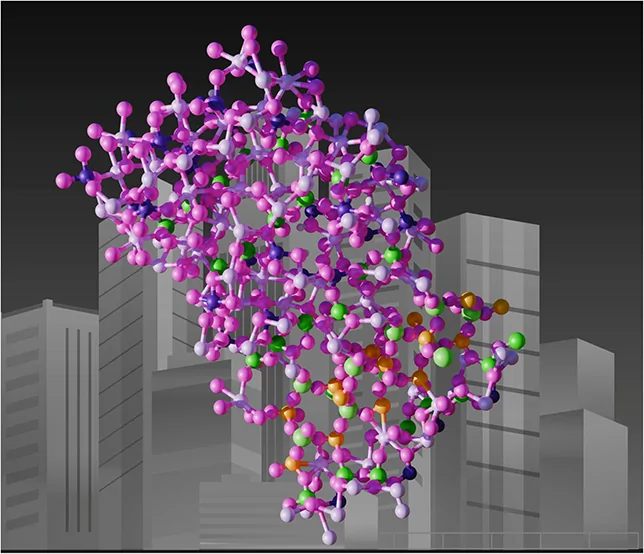

## 1 Introduction

The civil construction industry faces pressing sustainability challenges due to the high carbon emissions associated with conventional cement production.^1–3^ Nevertheless, geopolymers have emerged as promising alternatives, offering reduced environmental impact and efficient utilization of aluminosilicate resources such as fly ash, metakaolin, and slag.^4–8^ Their inherent chemical versatility and lower carbon footprint make them attractive candidates for next-generation construction materials. Geopolymers are inorganic polymeric systems composed of interconnected −Si–O–Al–O– frameworks that impart excellent mechanical strength and durability.^9–14^ The geopolymerization process involves the dissolution of aluminosilicate precursors, releasing tetrahedral Si^4+^ and Al^3+^ species that subsequently undergo polycondensation to form a three-dimensional network. Among various supplementary cementitious materials (SCMs), metakaolin stands out for its high chemical reactivity, a trait driven by the amorphous structure that it gains during kaolinite dehydroxylation. Under alkaline conditions, hydroxyl ions attack the Si–O–Si and Si–O–Al bonds, causing the progressive dissolution of silicate and aluminate species. The resulting structural stability is strongly governed by the Si/Al ratio and the presence of charge-balancing cations, which regulate the framework connectivity and electrostatic interactions. Atomistic investigations employing density functional theory (DFT), machine-learning force fields (MLFFs), and the improved dimer method (IDM) have delivered molecular-level insights into the dissolution mechanisms of metakaolin.^15^ Optimized compositions promote robust network formation, whereas excess alkali content can disrupt structural integrity.

Recent studies show that nanoscale fillers in geopolymer matrices can significantly improve fracture toughness, reduce brittleness, and suppress crack propagation, thereby enhancing overall performance.^16–25^ Two-dimensional (2D) materials have emerged as particularly effective reinforcements due to their high surface area, tunable surface chemistry, and strong interactions with the geopolymer network.^26–39^ Graphene, a single layer of sp^2^ hybridized carbon atoms arranged in a honeycomb lattice, is the most extensively studied 2D material and exhibits exceptional mechanical properties, including high stiffness, strength, and toughness.^37,40,41^ However, pristine graphene is inherently hydrophobic and chemically inert, which limits its dispersion and interfacial bonding within geopolymer matrices. To overcome these limitations, surface functionalization is widely employed to introduce chemically active groups onto graphene. However, in graphene derivatives such as graphene oxide (GO) and reduced graphene oxide (RGO), the interface is dominated by oxygen-containing groups (−OH, −COOH, and epoxy) that form hydrogen bonds and electrostatic interactions with surface hydroxyls and Ca^2+^ ions, with optimized structures showing larger interfacial distances indicative of moderate binding strength.^25,42–45^ However, functionalization plays a critical role in enhancing the dispersion and interfacial interactions of graphene derivatives in geopolymer matrices. Recently, our group reported that 2D derivatives such as sulfonated graphene (G-SO_3_H), thiographene (G-SH), and phosphate-functionalized graphene (G-PO_3_H) interact strongly with the matrix through hydrogen bonding, electrostatic interactions, and ion-mediated bridging with alkali and alkaline earth cations.^46^ Experimental characterization and DFT calculations confirm that these interactions promote robust interfacial adhesion, improving particle distribution, matrix density, and overall mechanical performance, highlighting their potential as effective reinforcements for high-performance geopolymer composites.^45^ Further, our recent work on Ti_3_C_2_(OH)_2_ MXene has demonstrated that surface-terminated 2D materials can provide strong interfacial adhesion through hydrophilic functional groups, highlighting the importance of surface chemistry in governing composite performance.^47^ However, these materials exhibit distinct electronic structures and surface chemistries, which critically influence their interaction with geopolymer binding phases such as calcium aluminosilicate hydrate (C-A-S-H). In this study, we systematically investigate a diverse class of materials, including Xenes, transition metal dichalcogenides (TMDs), MXenes, graphene derivatives, and ceramic/covalent systems, to establish a fundamental understanding of their interfacial behavior. The interactions are governed by a combination of covalent bonding, ionic coordination, electrostatic attraction, hydrogen bonding, and van der Waals forces. Xenes such as silicene and germanene exhibit strong covalent interactions with C-A-S-H, reflected in high interaction energies (−56.26 and −41.01 eV), due to their mixed sp^2^/sp^3^ hybridization. In contrast, transition metal dichalcogenides show moderate interactions driven by Ca–S coordination and charge transfer, while functionalized MXenes and graphene derivatives interact through surface functional groups via electrostatic and ion-bridging mechanisms. Moreover, ceramic systems such as silicon carbide (−12.44 eV) and boron phosphate (−31.65 eV) exhibit strong interfacial bonding, whereas materials like boron nitride and CrO_2_ show comparatively weaker interactions. These variations highlight the importance of surface chemistry and electronic structure in governing interfacial strength. Therefore, this work provides a unified framework for designing next-generation geopolymer composites with enhanced performance and durability.

## 2 Methodology

Periodic calculations were carried out using the Vienna ab initio Simulation Package (VASP) implementation of DFT within the projector augmented-wave (PAW) framework.^48,49^ The simulations were performed at the PBE-D3 level of theory.^50,51^ A plane-wave cutoff energy of 400 eV was employed. The k-point sampling grid was generated using the Gamma-point scheme with a 1 × 1 × 1 k-point mesh. The positions of all atoms were fully optimized with an electronic energy convergence threshold of 10^–6^ eV.

### 2.1 Model Construction

The structural model of C-A-S-H was generated based on the crystal structure of 14 Å tobermorite, which is widely used as a structural analogue for C-S-H gels.^52,53^ Substitution of a Si atom with an Al atom introduces a negative charge into the structure. To maintain charge neutrality and ensure chemical stability, one proton was therefore added to the unit cell. In previous studies, charge compensation was achieved by substituting a Si atom with an Al atom and simultaneously replacing an interlayer Ca atom with an alkali metal, with the resulting charge imbalance compensated by the addition of two protons. A similar charge-balancing approach was adopted in recent studies.^54^ However, for simplicity, alkali-metal ions were not included in the model, and charge neutrality was achieved solely by introducing one proton per Al substitution. In this study, the Ca/Si ratio was maintained at approximately 1.4, while the Ca/(Si+Al) ratio was set to 1.0. These values were selected because slag-based alkali-activated materials (AAMs), which predominantly contain C-(N)-A-S-H gel, typically exhibit Ca/Si and Ca/(Si+Al) ratios within this range.^55–57^ Cell volumes (a × b × c) used in geometry optimization for each investigated hybrid system are listed in Table S1.

The total interaction energy (Δ*E*, eq 1) was evaluated as the energy difference between the complex and its isolated components—namely, C-A-S-H and the 2D material. This corresponds to the binding process and is expressed as

1 $$\Delta E=E_{complex}-E_{\mathrm{C-A-S-H}}-E_{2D\;material}$$

Further, the total interaction energies were normalized by surface area.

We performed Bader charge analysis to compute the total charge associated with each atom;^58,59^ thus, charge-transfer values were calculated based on this analysis.

*Ab initio* molecular dynamics (MD) simulations were performed in the isothermal-isobaric (NPT) ensemble until equilibrium was reached. The MD simulations were performed at 300 K using a Langevin thermostat at standard pressure with a time step of 1 fs.

## 3 Results and Discussion

2D materials have attracted significant attention because of their unique physicochemical properties, including high surface area, tunable surface chemistry, and exceptional mechanical and electronic characteristics. In particular, different 2D material families, including Xenes, MXenes, graphene derivatives, transition metal dichalcogenides, carbides, and ceramic 2D systems, offer distinct interaction mechanisms such as hydrogen bonding, electrostatic attraction, ion-mediated bridging, and van der Waals forces, as shown in Scheme 1. These interactions directly influence the nucleation, gel growth, and microstructural evolution within the geopolymer matrix. Therefore, a DFT-guided and simulation-supported study of these materials can provide fundamental insights into the interfacial behavior, nucleation processes, and gel evolution in geopolymer systems. Such a systematic framework is essential for developing a unified understanding of structure-property relationships and for enabling the rational design of 2D-material-reinforced geopolymers with improved mechanical properties.

**Scheme 1:**
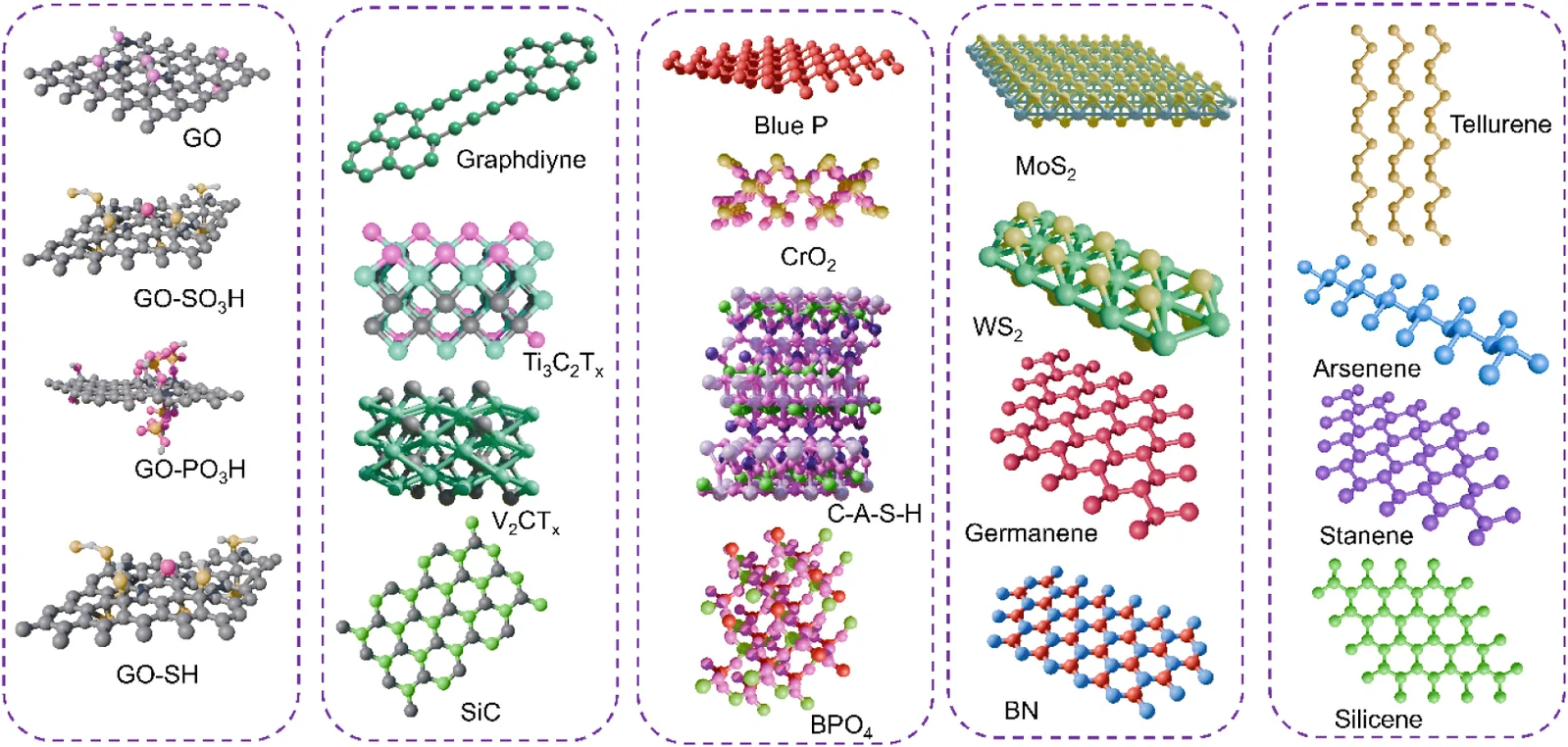
Schematic of Representative Two-Dimensional (2D) Material Families-Graphene Derivatives, Carbon Allotropes, MXenes, Carbides, Oxides, Dichalcogenides, Ceramics, and Xenes Highlighting their Structural Diversity and Relevance to Interfacial Interactions in Geopolymer Systems (C-A-S-H)

### 3.1 Graphene Derivatives

Graphene derivatives play a crucial role in enhancing the performance of geopolymer systems due to their high surface area, tunable surface chemistry, and strong interfacial interaction capabilities. In particular, graphene oxide (GO) has been widely studied using computational chemistry methods to understand its interaction with cementitious phases such as C-S-H, C-A-S-H, and N-A-S-H. To elucidate the reinforcement mechanisms of graphene derivatives in C-A-S-H-based geopolymer systems, density functional theory (DFT) calculations were performed on model interfaces between functionalized graphene sheets and C-A-S-H surfaces (Figure 1a). Geometry optimization revealed that all graphene derivatives adopt stable configurations on the C-A-S-H substrate, with distinct interfacial bonding characteristics depending on the surface functional groups. In the case of graphene oxide (GO), the interface is governed by abundant oxygen-containing functionalities, which form hydrogen bonds and electrostatic interactions with surface hydroxyl groups and charge-compensating Ca^2+^ ions. The optimized structures show relatively larger interfacial distances compared to other derivatives, indicative of moderate binding strength. This type of interaction facilitates homogeneous dispersion within the C-A-S-H-based geopolymer matrix and contributes to pore refinement. For thiographene (G-SH), the presence of thiol groups introduces localized covalent interactions and weak coordination bonds with surface Ca atoms (Figure 1b).

**Figure 1:**
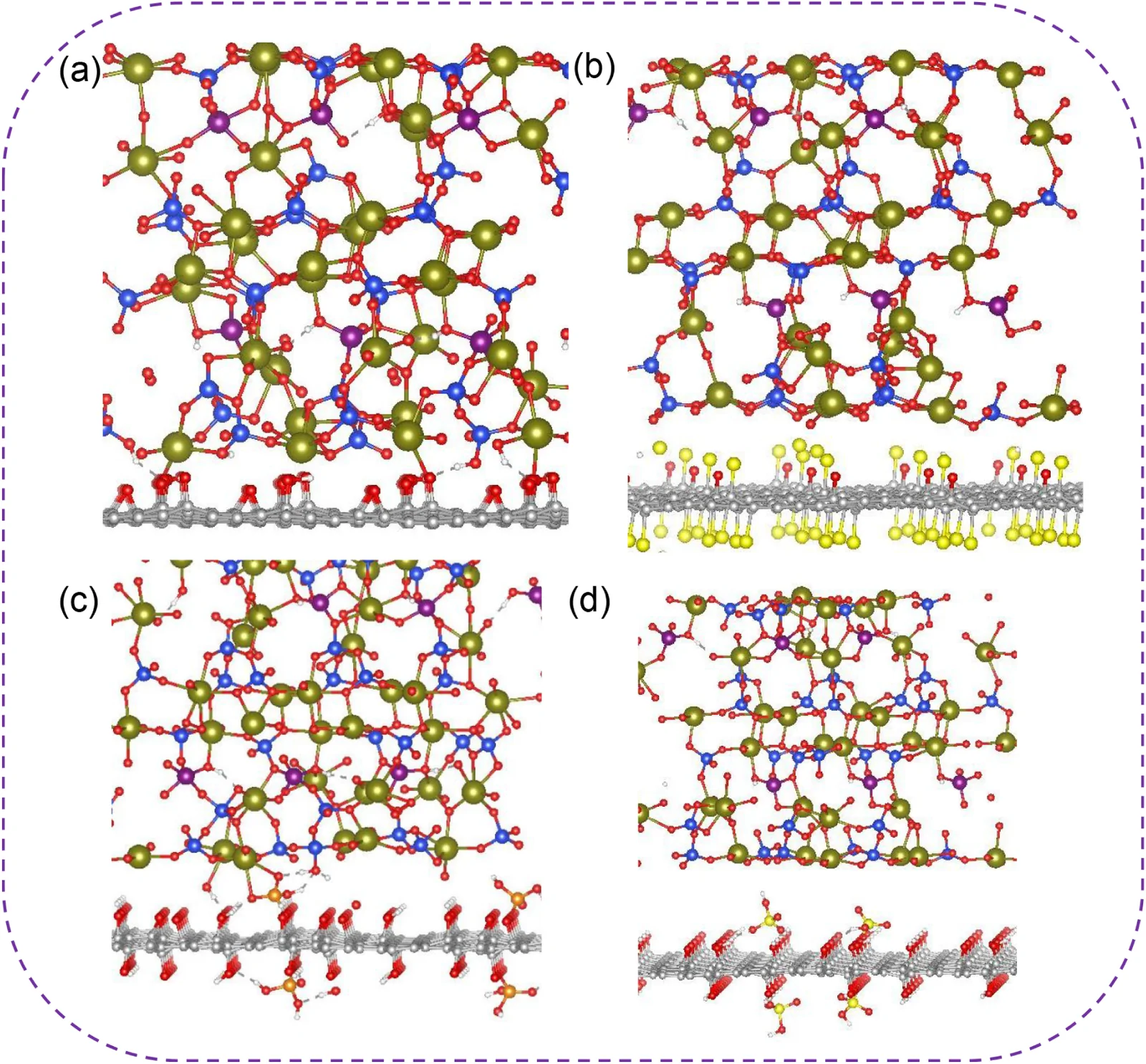
Atomistic models illustrating interfacial configurations of graphene derivatives on C-A-S-H—(a) GO, (b) G-SH, (c) G-PO_3_H, and (d) G-SO_3_H modifies interfacial bonding, structural arrangement, and interaction strength with the C-A-S-H surface. [Calcium: gold, Oxygen: red, Silicon: blue, Aluminum: purple, Carbon: gray, Hydrogen: white, Phosphorus: orange, Sulfur: yellow].

The optimized geometry suggests partial charge transfer from sulfur atoms to Ca sites, resulting in stronger anchoring than with GO, while maintaining structural flexibility. This balance is particularly beneficial for enhancing durability and chemical resistance under harsh conditions. Phosphate-functionalized graphene (G-PO_3_H) exhibits the strongest interfacial interaction among all systems (Figure 1c). The optimized structures clearly show the formation of P–O–Ca linkages, arising from ion-exchange processes and covalent bonding with the substrate. These interactions significantly reduce the interfacial separation and lead to a highly stable hybrid structure, suggesting efficient stress transfer and strong adhesion at the interface. In contrast, sulfonated graphene (G-SO_3_H) demonstrates dominant ionic interactions between deprotonated −SO_3_^–^ groups and Ca^2+^ ions on the C-A-S-H (Figure 1d). The optimized configurations reveal multiple anchoring points, forming a dense interfacial network that enhances structural cohesion and promotes rapid nucleation during geopolymerization. Functionalization with thiol groups (G-SH) enhances the interaction strength through partial covalent character and weak coordination bonding with surface Ca atoms. The highest interaction energy is obtained for G-PO_3_H, reflecting the synergistic contribution of ion-exchange processes and the formation of robust covalent P–O–Ca linkages. To gain deeper insight into the interfacial bonding mechanisms, charge-density-difference and electronic-structure analyses were performed (Figure S1). In GO-based systems, charge redistribution is relatively delocalized across the interface, consistent with the predominance of weak electrostatic and hydrogen-bonding interactions. In contrast, the G-SH system exhibits localized charge accumulation at the S–Ca interaction sites, confirming the presence of coordination bonding with partial covalent character. For G-SO_3_H, a significant transfer of electron density from sulfonate groups to Ca centers is observed, indicative of strong ionic interactions that stabilize the interface (Figure S1). The most pronounced charge redistribution occurs in the G-PO_3_H system, where clear directional electron density overlaps between P–O groups and Ca atoms confirm the formation of partially covalent bonds. This strong electronic coupling is consistent with the highest calculated interaction energy and superior interfacial stability.

Another interesting 2D graphene derivative, Graphdiyne, is a unique carbon allotrope composed of sp- and sp^2^-hybridized carbon atoms connected via diacetylenic linkages (−C≡C–C≡C−), exhibiting a highly conjugated two-dimensional framework with intrinsic, uniformly distributed nanopores. This distinctive structure enables a combination of extended π-electron delocalization and inherent porosity, distinguishing it from conventional graphene-based materials. The DFT-optimized Graphdiyne@C-A-S-H interface reveals that the interaction is primarily governed by π–surface interactions and porous adsorption mechanisms, with additional weak electrostatic contributions involving surface Ca^2+^ ions (Figure 2). The partial density of states (PDOS) further indicates a contribution from the C 2p orbitals in the valence-band region, although their contribution is relatively small (Figure S2). The planar Graphdiyne sheet aligns parallel to the C-A-S-H surface, maximizing interfacial contact and facilitating cation-π-type interactions with the silicate/aluminate network. Simultaneously, its intrinsic pores act as active sites for adsorption of ions and water molecules, enhancing interfacial functionality. The calculated interaction energy for the Graphdiyne@C-A-S-H system is E = −4.35 eV, indicating a moderately strong interaction regime.

**Figure 2:**
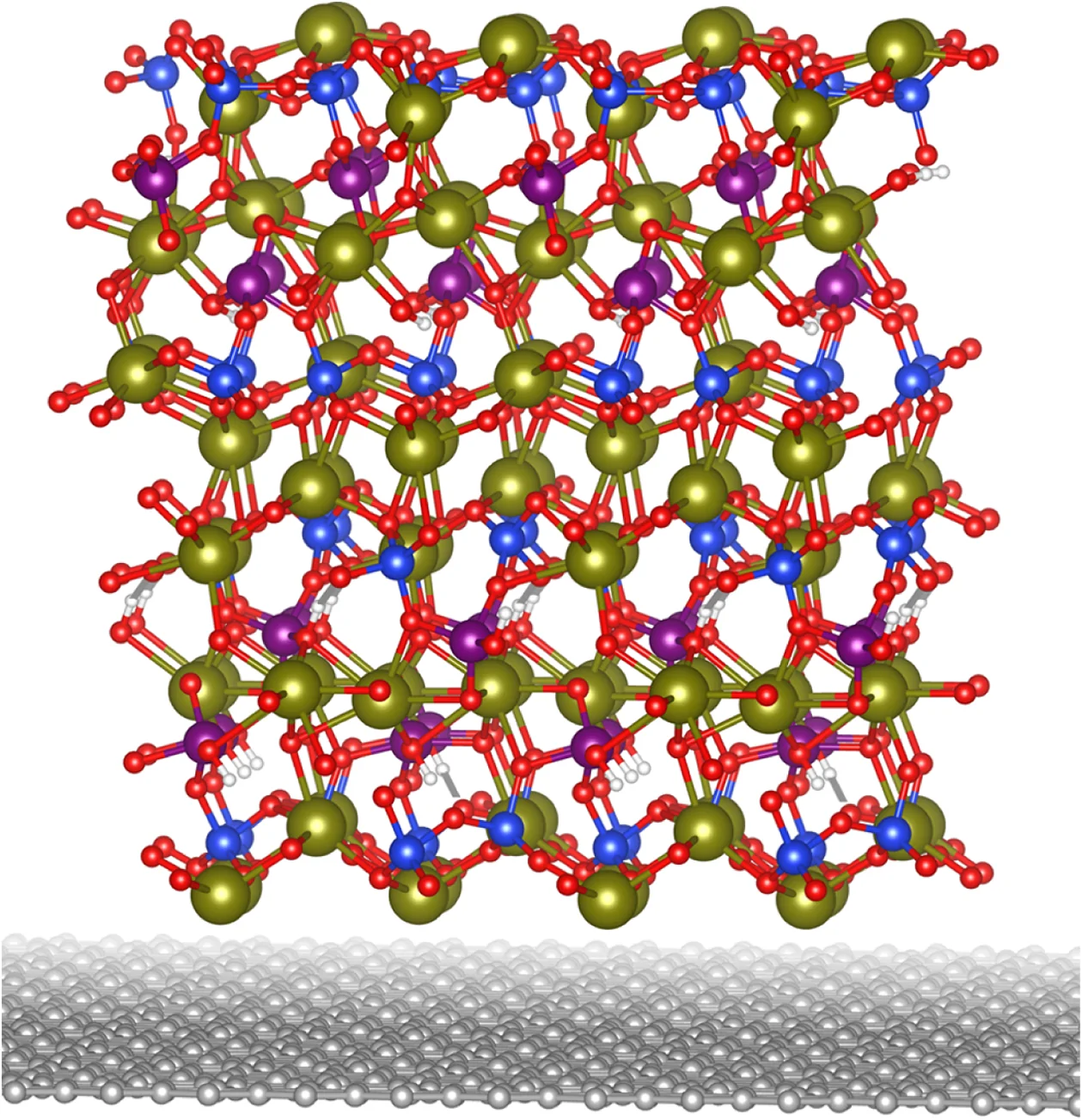
DFT-optimized structure of the Graphdiyne@C-A-S-H interface, showing a graphdiyne layer interacting with the C-A-S-H surface primarily through π–surface interactions. [Calcium: gold, Oxygen: red, Silicon: blue, Aluminum: purple, Carbon: gray, Hydrogen: white].

This value is significantly higher than typical van der Waals interactions and comparable to strongly adsorbed 2D materials, yet remains lower than systems dominated by covalent bonding. Such an optimal interaction strength ensures sufficient interfacial adhesion while preserving structural flexibility and transport pathways. Charge density difference analysis indicates delocalized electron redistribution across the π-conjugated network, with minor charge accumulation near Ca sites, confirming the predominance of non-covalent interactions (Figure S1). This is further evidenced by the Bader charge analysis, which reveals only minimal charge transfer (−0.07e) in Graphdiyne@C-A-S-H. In general, DFT results demonstrate that tailoring interfacial interactions in graphene-based systems directly enhances C-A-S-H-based geopolymer strength, durability, and overall performance. Functionalized graphene (GO, G-SH, G-SO_3_H, G-PO_3_H) improves dispersion, bonding, and reaction kinetics, leading to higher compressive strength, reduced porosity, and better thermal and chemical stability. In contrast, Graphdiyne introduces π-interaction-driven adsorption and ion transport, further improving mass transfer and matrix densification. Overall, these materials enable the design of high-performance geopolymers for structural, high-temperature, and environmental applications.

### 3.2 MXene Derivatives

MXenes are an emerging class of 2D transition metal carbides and nitrides that have garnered significant attention as promising counterparts to graphene.^60–63^ Synthesized via selective etching of the “A” layer from MAX phases, MXenes possess surface terminations (−OH, −F) that critically dictate their physicochemical properties. They exhibit a unique combination of high electrical conductivity, intrinsic hydrophilicity, large surface area, tunable surface chemistry, and electron-rich character, along with excellent mechanical strength and flexibility. Importantly, their scalable and controllable synthesis makes them highly suitable for large-scale applications.

Recently, our group reported the incorporation of MXene (Ti_3_C_2_Tx T_*x*_ = −OH, −F) into C-A-S-H-based geopolymer systems, revealing a synergistic interaction mechanism governed by intermolecular hydrogen bonding and ion bridging (Ca^2+^) at the interface. Hydrogen bonds (O–H···O) form with the hydroxylated oxygen atoms of the silicate chain, with a characteristic hydrogen bond length of 1.633 Å. These interactions promote the formation of a dense and well-integrated matrix, resulting in enhanced mechanical strength and improved electrical conductivity (Figure 3a and Figure S3). Furthermore, DFT calculations highlighted the critical role of hydrogen bonding in strengthening Ti_3_C_2_Tx@C-A-S-H interactions. As a result, the MXene-reinforced system exhibited a significant improvement in mechanical performance, with compressive strength increasing by approximately 53%, accompanied by a 3.6% reduction in water absorption, attributed to the formation of a compact and less porous microstructure.

**Figure 3:**
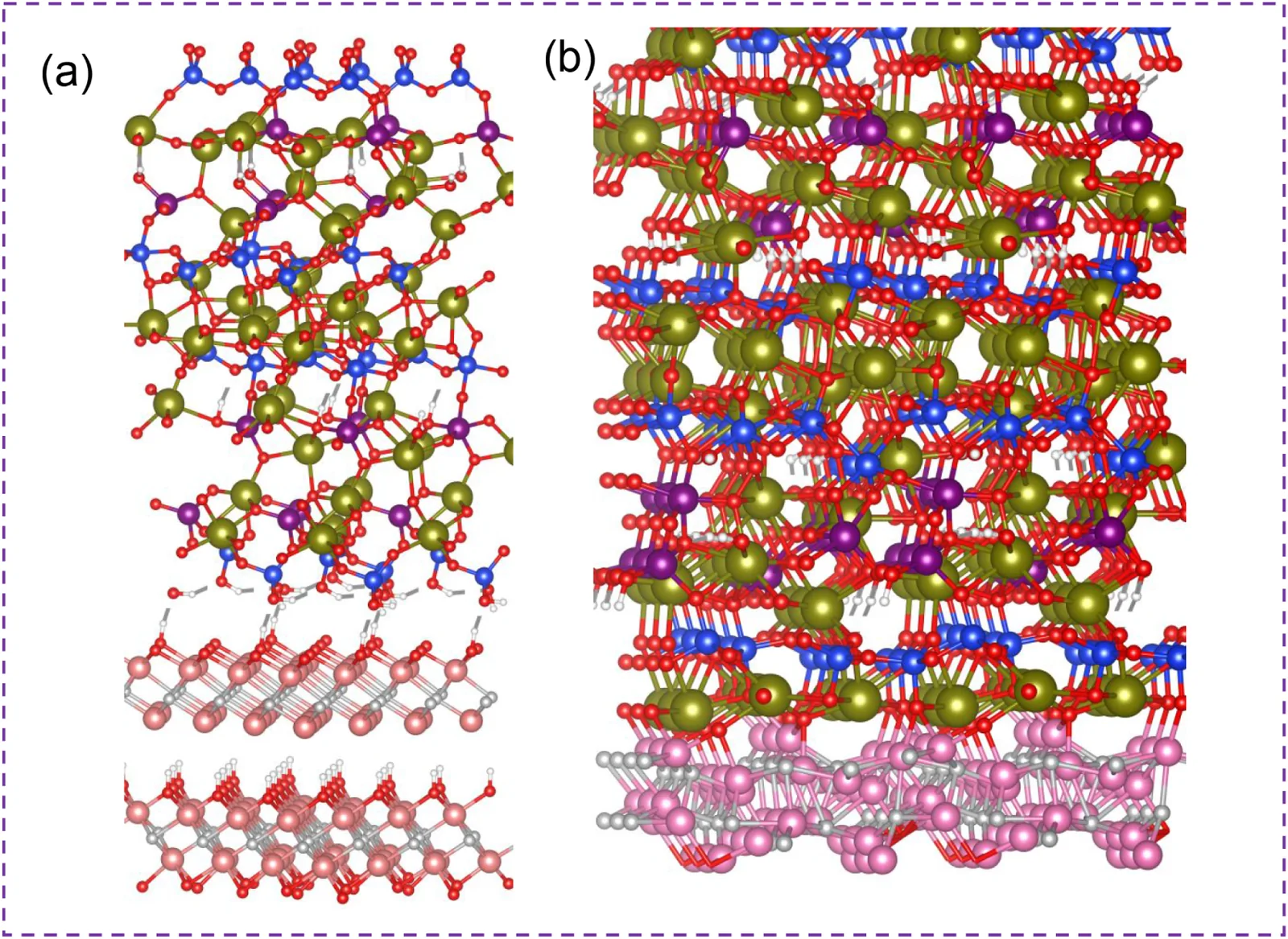
DFT-optimized structures of (a) Ti_3_C_2_Tx (−OH, −F) and (b) V_2_C MXenes on the C-A-S-H surface, showing strong interfacial interactions. [Calcium: gold, Oxygen: red, Silicon: blue, Aluminum: purple, Titanium: pink, Vanadium: bright pink, Carbon: gray, Hydrogen: white].

Vanadium carbide (V_2_C) holds significant potential as an advanced reinforcement material for C-A-S-H concrete due to its ability to establish covalent and metallic interactions at the interface (Figure 3b). When the C-A-S-H model interacts with V_2_C, the oxygen atoms of the silicate chain coordinate with the vanadium atoms at the surface. The bonding arises from orbital interactions between the O 2p orbitals and the V 3d orbitals. Specifically, the metal donates electron density to the oxygen π* orbitals, while the oxygen simultaneously donates electron density from its filled p orbitals into the partially filled 3d states of vanadium, resulting in π back-donation. This charge transfer leads to the formation of a strong interfacial bond between the C-A-S-H model and V_2_C. The calculated interaction energy −44 eV indicates a robust binding regime, significantly stronger than purely physisorbed systems. Charge density analysis confirms substantial electron redistribution at the interface, highlighting the dual covalent-metallic nature of bonding (Figure S3). Overall, MXenes’ unique ability to establish diverse interfacial interactions, including hydrogen bonding, ion bridging, covalent bonding, and metallic electron coupling, enables significant improvements in mechanical strength, durability, and electrical conductivity. These interactions promote the formation of dense, well-integrated microstructures with reduced porosity and enhanced load transfer efficiency. Importantly, the tunable surface chemistry and intrinsic hydrophilicity of MXenes ensure excellent dispersion within aqueous C-A-S-H matrices, overcoming limitations associated with conventional carbon-based additives.

### 3.3 Transition Metal Dichalcogenides (TMDs)

TMDs such as MoS_2_ and WS_2_ have emerged as prototypical two-dimensional systems owing to their intrinsic bandgaps and pronounced light-matter interactions.^27,30,31,64^ In this context, their integration with cementitious matrices, specifically C-A-S-H, opens an unexplored pathway toward multifunctional structural materials. Structurally, TMD monolayers consist of a transition-metal layer sandwiched between two chalcogen (S) layers, resulting in chemically inert basal planes dominated by outward-facing sulfur atoms. These surface sulfur atoms possess lone pairs of electrons, which, while contributing to the intrinsic chemical stability of TMDs, also enable selective interfacial interactions in ionic environments. Within the C-A-S-H framework, these sulfur sites act as electron donors and coordinate with Ca^2+^ ions, establishing interfacial binding governed by electrostatic attraction and coordination chemistry.

To elucidate the thermodynamic stability of these hybrid interfaces, DFT calculations were performed. The computed interaction energy for the MoS_2_@C-A-S-H interface is −2.01 eV, indicating a strongly favorable binding configuration (Figure 4a). This negative interaction energy suggests spontaneous adsorption, driven by Ca–S coordination and charge redistribution at the interface. However, the WS_2_@C-A-S-H system exhibits an interaction energy of +2.88 eV, implying comparatively weaker interfacial stabilization under similar conditions (Figure 4b). The disparity in interaction energies can be attributed to differences in electronic structure and orbital overlap between Mo–S and W–S systems, influencing their affinity toward Ca^2+^ coordination. Charge density analysis indicates that electron redistribution is predominantly localized within the WS_2_ surface rather than at the interface (Figure S4). The Bader charge analysis further supports this observation, revealing minimal charge transfer in the WS_2_@C-A-S-H system, whereas substantial charge transfer (−0.26 e) occurs in the MoS_2_@C-A-S-H system. The PDOS further reveals a contribution from the Mo 4d orbitals in the valence-band region, with a more pronounced contribution in the conduction-band region (Figure S5). From a mechanistic standpoint, the incorporation of MoS_2_ into the C-A-S-H matrix is expected to promote enhanced interfacial adhesion through Ca^2+^ bridging, leading to improved load transfer and matrix densification. Additionally, the intrinsic van der Waals (VdW) sliding nature of MoS_2_ layers contributes a solid lubrication effect, which can mitigate microcrack propagation and enhance fracture resistance. This dual functionality-chemical bonding combined with mechanical energy dissipation-positions MoS_2_ as a highly effective nanoscale reinforcement in C-A-S-H systems. On the other hand, WS_2_, while exhibiting comparatively weaker binding energetics, offers distinct advantages in terms of surface adsorption stability and oxidation resistance, making it suitable for applications in chemically aggressive or high-temperature environments. Its durability may still contribute to long-term stability, albeit with a reduced interfacial strengthening effect compared to MoS_2_. These findings provide a fundamental basis for future experimental validation and open new directions for integrating 2D materials into sustainable construction materials.

**Figure 4:**
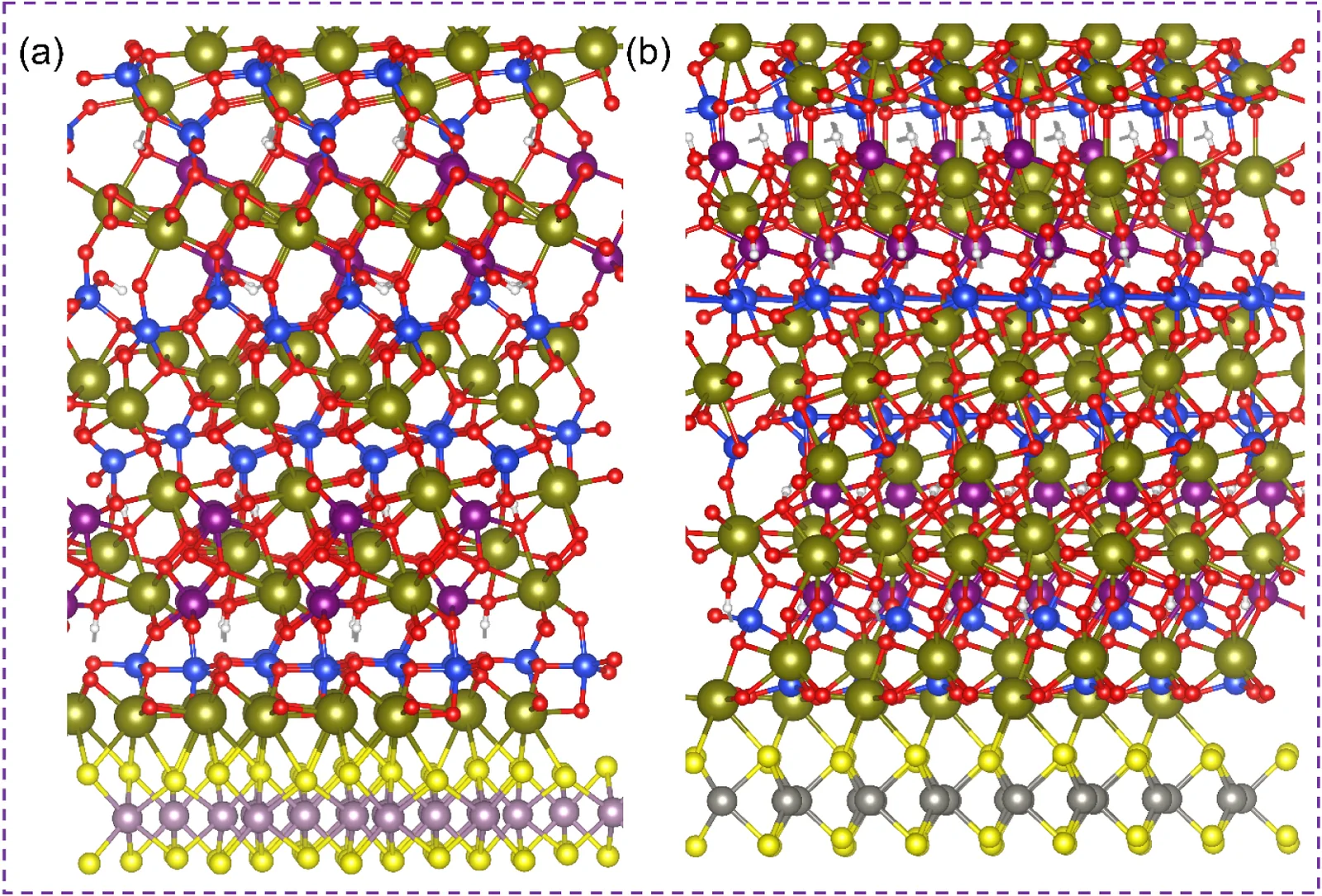
DFT analysis of the C-A-S-H interaction with (a) MoS_2_ and (b) WS_2_. shows strong Ca–S bonding and charge transfer for MoS_2_ (−2.01 eV), while WS_2_ exhibits weaker interaction (+2.88 eV). [Calcium: gold, Oxygen: red, Silicon: blue, Aluminum: purple, Sulfur: bright yellow, Molybdenum: lavender, Tungsten: dark gray, Hydrogen: white].

### 3.4 2D-Derived Inorganic Materials

Among 2D-derived inorganic materials, silicon carbide (SiC) has emerged as a particularly attractive candidate due to its exceptional chemical stability, wide bandgap, and robust electronic properties that persist even under ambient conditions.^65–68^ Unlike intrinsically layered materials, SiC adopts a three-dimensional covalent framework. However, when engineered in ultrathin or surface-dominated forms, it exhibits interfacial characteristics analogous to 2D systems. In particular, the cubic polytype 3C-SiC crystallizes in a zinc-blende structure and behaves as a wide-bandgap semiconductor with high thermal and chemical resilience. In this study, a Si-terminated SiC surface is considered to investigate its interaction with the C-A-S-H model (Figure 5a). From a chemical perspective, the surface-exposed Si atoms possess partially unsaturated coordination, rendering them highly reactive toward oxygen-containing species. Upon contact with the C-A-S-H matrix, these Si sites readily interact with silanol (Si–OH) groups present in the hydrated aluminosilicate network, leading to the formation of Si–O–Si covalent linkages at the interface. This condensation-type reaction is driven by the strong affinity of silicon for oxygen, resulting in the release of interfacial strain and the stabilization of the hybrid structure. DFT calculations further confirm the strength of this interaction, with a calculated interaction energy of −12.44 eV, indicating highly favorable and robust interfacial bonding. This value is significantly more negative than those observed for typical van der Waals or coordination-driven systems, highlighting the dominance of covalent bond formation at the SiC@C-A-S-H interface. Such a strong chemical coupling is expected to substantially enhance load transfer, reduce interfacial defects, and improve the overall mechanical integrity of the composite. The interaction mechanism in SiC differs fundamentally from that of layered 2D materials. While materials such as dichalcogenides rely on electrostatic interactions or weak coordination bonding, SiC establishes a direct covalent bridge with the silicate network, effectively integrating into the C-A-S-H framework at the atomic level (Figure S6). The PDOS analysis shows the presence of C 2p orbital contributions in the valence band region (Figure S7). This distinction positions SiC as a highly promising reinforcement phase for C-A-S-H systems, where strong interfacial bonding is critical to achieving superior durability and structural performance.

**Figure 5:**
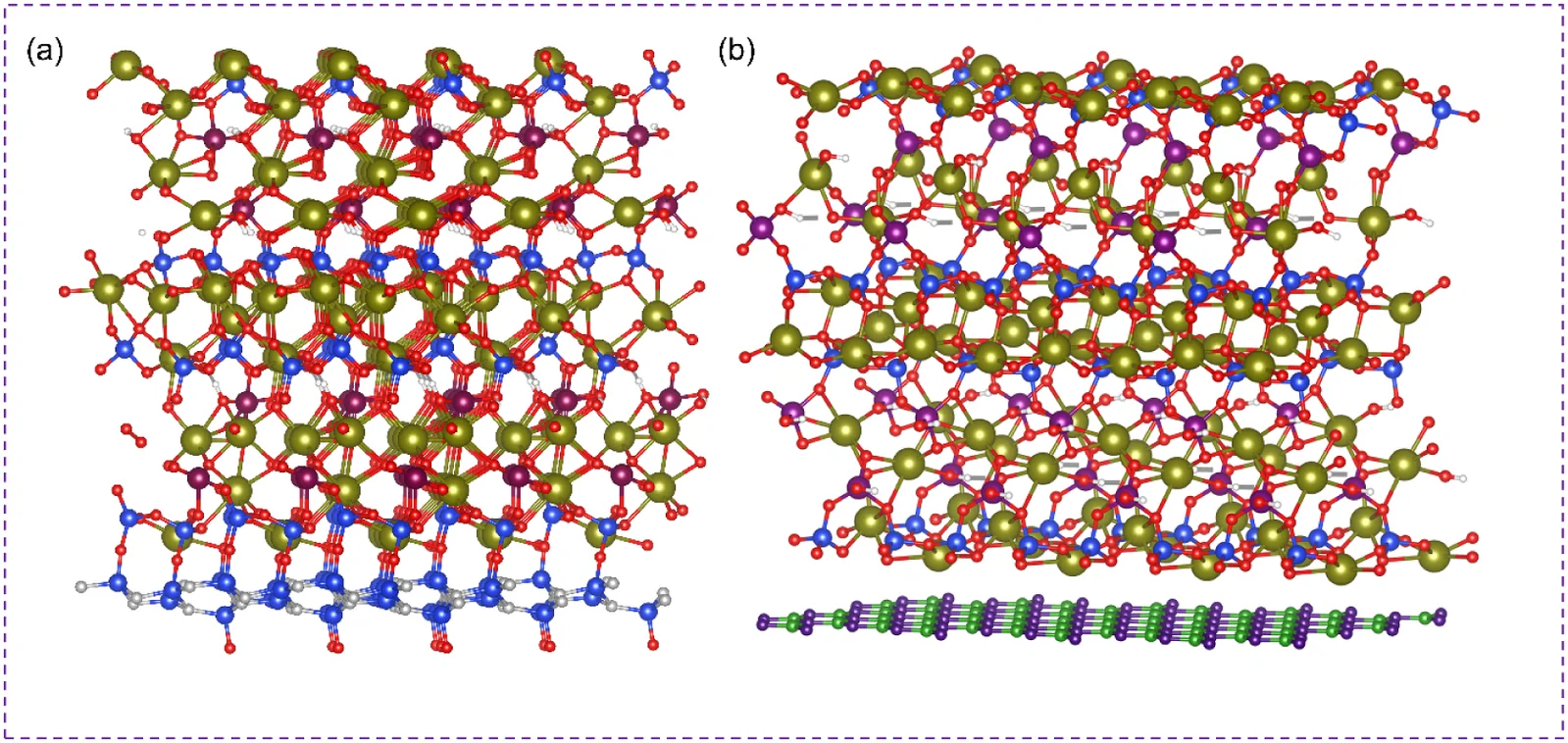
DFT analysis of C-A-S-H interaction with (a) silicon carbide and (b) h-BN shows strong Si–O–Si covalent bonding and significant interfacial stabilization for SiC@C-A-S-H, with a highly favorable interaction energy of −12.44 eV, while h-BN shows weak interactions dominated by non-covalent interactions (−1.45 eV). [Calcium: gold, Oxygen: red, Silicon: blue, Aluminum: purple, Carbon: light gray, Boron: green, Nitrogen: violet, Hydrogen: white].

When the C-A-S-H model interacts with h-BN, Ca^2+^ ions from the C-A-S-H phase are retained at the interfacial region, where they engage with the electron-rich surface of h-BN (Figure 5b). This interaction promotes interfacial stabilization and improves the overall structural integrity of the hybrid system. Charge density analysis indicates that minimal electron redistribution is localized at the interface (Figure S6). The Bader charge analysis is consistent with this observation, indicating negligible charge transfer (−0.014 e) in the BN@C-A-S-H system. The PDOS analysis further indicates that the B 2p and N 2p orbitals are primarily located at deeper valence-band energies, with negligible contributions near the Fermi level (Figure S7). Such non-covalent interactions provide relatively lower stabilization compared to direct covalent bonding, as observed in systems involving MXene surfaces. The calculated interaction energy of −1.45 eV further supports the presence of moderate interfacial interactions.

Another 2D-derived inorganic material, borophosphate (BPO_4_), is known for its rigid covalent framework and high chemical stability.^69–71^ Although BPO_4_ is not intrinsically layered, its surface-terminated structures exhibit chemically active oxygen sites that enable strong interfacial interactions with hydrated cementitious phases. In the BPO_4_@C-A-S-H system, the oxygen atoms within the B–O–P framework play a crucial role in interfacial bonding (Figure 6a). These oxygen atoms, possessing lone electron pairs, interact strongly with Ca^2+^ ions present in the C-A-S-H matrix. This leads to the formation of Ca–O coordination bonds, which anchor the BPO_4_ structure at the interface. Simultaneously, the covalent B–O–P network remains intact, providing structural rigidity. As a result, the interaction can be described as a combination of ionic (Ca^2+^–O) and covalent (B–O–P framework) contributions, leading to a highly stable hybrid interface.

**Figure 6:**
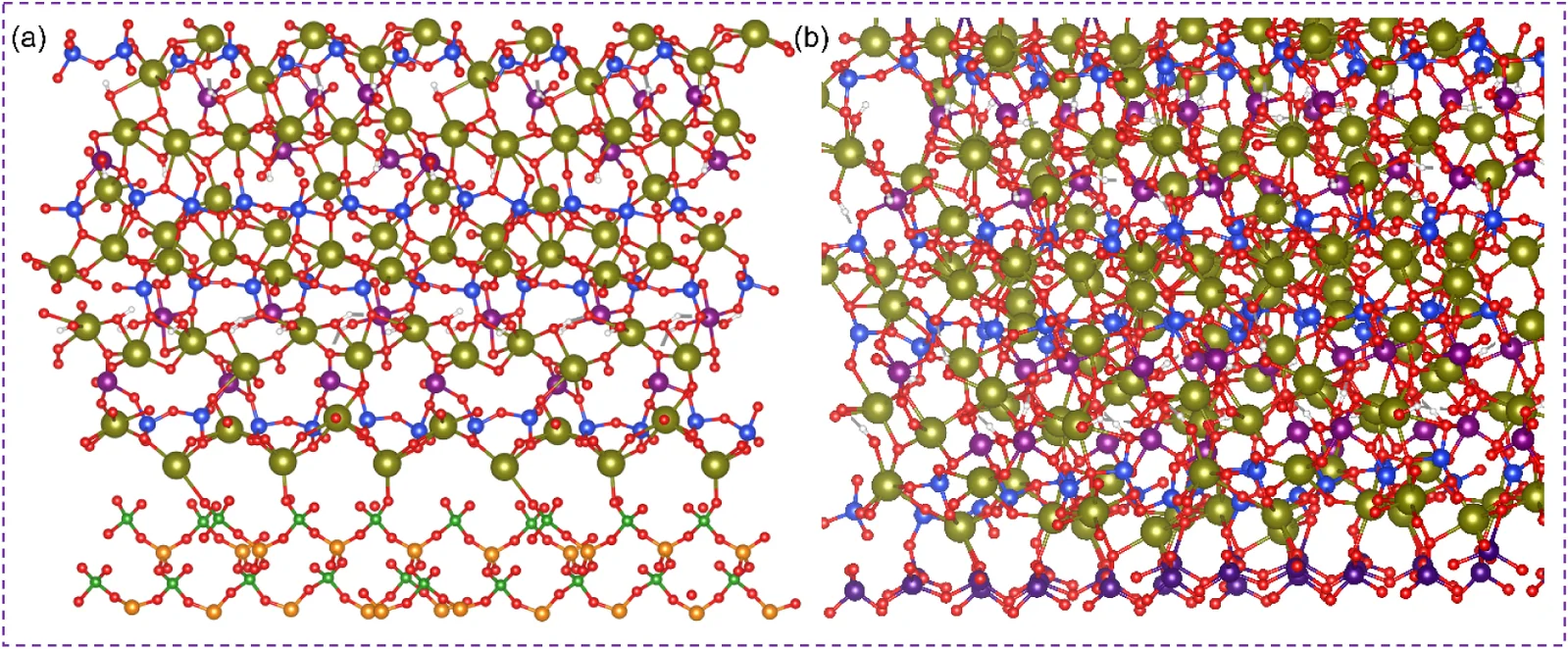
DFT analysis of C-A-S-H interaction with (a) boron phosphate and (b) CrO_2_. Results show very strong ionic–covalent Ca–O interactions for BPO_4_ (−31.65 eV), indicating high interfacial stability, while CrO_2_ exhibits comparatively moderate interaction dominated by weak coordination and electrostatic forces (−9.03 eV). [Calcium: gold, Oxygen: red, Silicon: blue, Aluminum: purple, Boron: green, Chromium: dark purple, Phosphorus: orange, Hydrogen: white].

From a chemical standpoint, this interaction is driven by the strong electrostatic attraction between electron-rich oxygen atoms and Ca^2+^ ions, along with partial charge transfer that enhances interfacial adhesion (Figure S8). The presence of multiple oxygen coordination sites further facilitates multi-point binding, significantly strengthening the interface compared to single-site interactions. DFT calculations reveal an exceptionally strong interaction energy of −31.65 eV, which is markedly more negative than that observed for other investigated systems, including silicon carbide. This large negative value indicates extremely favorable binding and suggests the formation of a densely integrated interfacial structure. The magnitude of this interaction energy highlights the dominant role of ionic–covalent coupling in stabilizing the BPO_4_@C-A-S-H system. Such strong interfacial bonding is expected to significantly enhance the mechanical integrity and durability of the composite by improving load transfer efficiency and reducing interfacial defects. Compared to systems governed primarily by weak van der Waals or coordination interactions, BPO_4_ exhibits a fundamentally different mechanism, where robust chemical bonding drives interface formation.

Another interesting 2D-derived inorganic material considered in this study is CrO_2_, a ferromagnetic transition metal oxide known for its high Curie temperature and unique electronic structure.^72–74^ CrO_2_ crystallizes in a rutile-type lattice, where Cr ions are arranged in a tetragonal framework and predominantly exist in the Cr^4+^ oxidation state, giving rise to its characteristic magnetic and conductive behavior. At the surface, oxygen atoms dominate the interfacial chemistry and serve as potential interaction sites with the C-A-S-H matrix (Figure 6b). Upon contact, these surface oxygen atoms can interact with Ca^2+^ ions or neighboring silicate species within the C-A-S-H structure. However, unlike strongly reactive systems such as silicon carbide or boron phosphate, the interactions in the CrO_2_ system are primarily governed by weak electrostatic attraction and limited coordination bonding, rather than the formation of robust covalent linkages. From a chemical perspective, the relatively lower reactivity arises from the already stabilized Cr–O framework, where oxygen atoms are less available for strong bond formation due to their involvement in the rutile lattice. As a result, interfacial interactions are restricted to surface adsorption and weak Ca–O coordination, without significant restructuring or bond formation across the interface. DFT calculations yield an interaction energy of −9.03 eV, indicating a thermodynamically favorable but comparatively weaker interaction than those observed for SiC and BPO_4_ systems. This intermediate interaction strength suggests partial stabilization of the interface, driven mainly by electrostatic interactions rather than strong chemical bonding. From a macroscopic standpoint, such moderate interfacial interaction may contribute to improved interfacial compatibility and dispersion within the C-A-S-H matrix, while preserving the intrinsic magnetic and electronic properties of CrO_2_. Unlike strongly bonded systems, CrO_2_ is less likely to chemically integrate into the silicate network, instead functioning as a functional additive that imparts additional properties without significantly altering the host structure. Among 2D-derived inorganic materials , boron phosphate exhibits the strongest interaction, driven by multi-site Ca–O ionic–covalent bonding, leading to highly stable and densely integrated interfaces. Silicon carbide, in contrast, promotes direct Si–O–Si covalent linkage with the silicate network, enabling effective structural integration and enhanced load transfer. Meanwhile, CrO_2_ shows comparatively moderate interaction, governed by weak coordination and electrostatic forces, making it suitable as a functional additive rather than a primary reinforcing phase. From a geopolymer concrete perspective, these distinct interaction mechanisms translate into different performance outcomes. BPO_4_ is expected to maximize matrix densification and interfacial strength, SiC contributes to mechanical reinforcement and structural continuity, while CrO_2_ offers functional properties such as electrical or magnetic responsiveness without significantly altering the matrix structure.

### 3.5 Xene-Based 2D Materials

Xene-based materials have attracted significant attention due to their unique structural and electronic properties. Representative members of this family include silicene, germanene, stanene, arsenene, tellurene, and blue phosphorus .^75–78^ Unlike graphene, which exhibits a perfectly planar geometry with pure sp^2^ hybridization, Xenes adopt a buckled honeycomb lattice, resulting from the competition between sp^2^ and sp^3^ hybridization. This intrinsic buckling plays a crucial role in defining their physicochemical behavior. It reduces lattice symmetry and introduces mixed π–σ bonding character, enhancing orbital overlap and creating chemically active surface sites. As a result, Xenes exhibit higher chemical reactivity and tunable surface functionality compared to graphene, making them particularly attractive for interfacial and composite applications. The presence of partially sp^3^-hybridized orbitals and electron-deficient regions further facilitates strong interactions with ionic or polar species.

In the first series, Group IV Xenes (silicene, germanene, and stanene) exhibit distinct interfacial behaviors governed by their hybridization and surface reactivity, as shown in Figure 7a–c. Among these, silicene shows the strongest interaction with C-A-S-H, with an interaction energy of −56.26 eV. The optimized structure reveals that oxygen atoms from the C-A-S-H matrix occupy bridging positions, forming multiple Si–O–Si covalent bonds, resulting in exceptionally strong interfacial coupling and structural integration (Figure 7a). Germanene also forms interfacial covalent linkages, specifically Si–O–Ge bonds, with a relatively high interaction energy of −41.01 eV (Figure 7b). However, due to its larger atomic radius and reduced orbital overlap compared to silicene, the interaction is comparatively less efficient. In contrast, stanene exhibits a significantly weaker interaction (−11.44 eV), with no clear formation of stable covalent bonds, indicating limited interfacial reactivity and predominantly weak adsorption behavior (Figure 7c). The study was further extended to Group V Xenes (pnictogens), including arsenene and blue phosphorus, as shown Figure 7d and 7f. These materials adopt buckled structures with predominant sp^3^ hybridization, where each atom possesses a localized lone pair oriented out of the plane. In arsenene, the presence of strongly localized non-bonding electron pairs limits electron delocalization and reduces the availability of electrons for interfacial bonding (Figure S9). Consequently, arsenene exhibits a moderate interaction energy of −20.73 eV, primarily governed by weak coordination and electrostatic interactions rather than strong covalent bonding (Figure 7d). The Bader charge analysis reveals a significant charge transfer of −3.29 e from arsenene to the C-A-S-H matrix. Similarly, blue phosphorus shows weaker interaction (−9.43 eV), reflecting limited charge transfer and reduced interfacial coupling (Figure 7f). The localized nature of lone-pair electrons in these systems restricts their ability to form strong chemical bonds with the C-A-S-H matrix. Tellurene exhibits an intermediate interaction energy of −14.95 eV, reflecting a balance between structural flexibility and moderate surface reactivity (Figure 7e). While it does not form strong covalent bonds like silicene, its heavier atomic character allows partial interaction through weak coordination and dispersion forces. The interaction strength of Xenes with the C-A-S-H matrix varies significantly across different materials. Silicene exhibits the strongest interaction with a binding energy of −56.26 eV, followed by germanene at −41.01 eV. Arsenene shows a moderate interaction of −20.73 eV, while tellurene and stanene display weaker interactions of −14.95 eV and −11.44 eV, respectively. Blue phosphorus has the lowest binding strength among the studied Xenes, with a value of −9.43 eV. This trend clearly demonstrates a transition from strong covalent interfacial bonding in Group IV Xenes to lone-pair-limited interactions in Group V systems. The exceptionally high interaction energies of silicene and germanene originate from their mixed sp^2^/sp^3^ hybridization, which provides accessible orbitals for the formation of stable Si–O–Si and Si–O–Ge covalent linkages with the C-A-S-H network. In contrast, the presence of localized lone-pair electrons in pnictogen-based Xenes restricts charge transfer and limits effective bonding, resulting in comparatively weaker interfacial interactions (Figure S9). From a geopolymer concrete perspective, these findings establish a direct structure–chemistry–performance relationship. Materials such as silicene and germanene, with strong covalent integration into the silicate framework, are expected to significantly enhance matrix densification, interfacial strength, and load transfer efficiency, thereby improving mechanical performance and durability. Conversely, more weakly interacting Xenes may contribute more to surface modification and functional tuning rather than structural reinforcement. Overall, this study highlights that tailoring hybridization and electronic structure at the nanoscale is key to designing next-generation geopolymer concretes with optimized mechanical and multifunctional properties.

**Figure 7:**
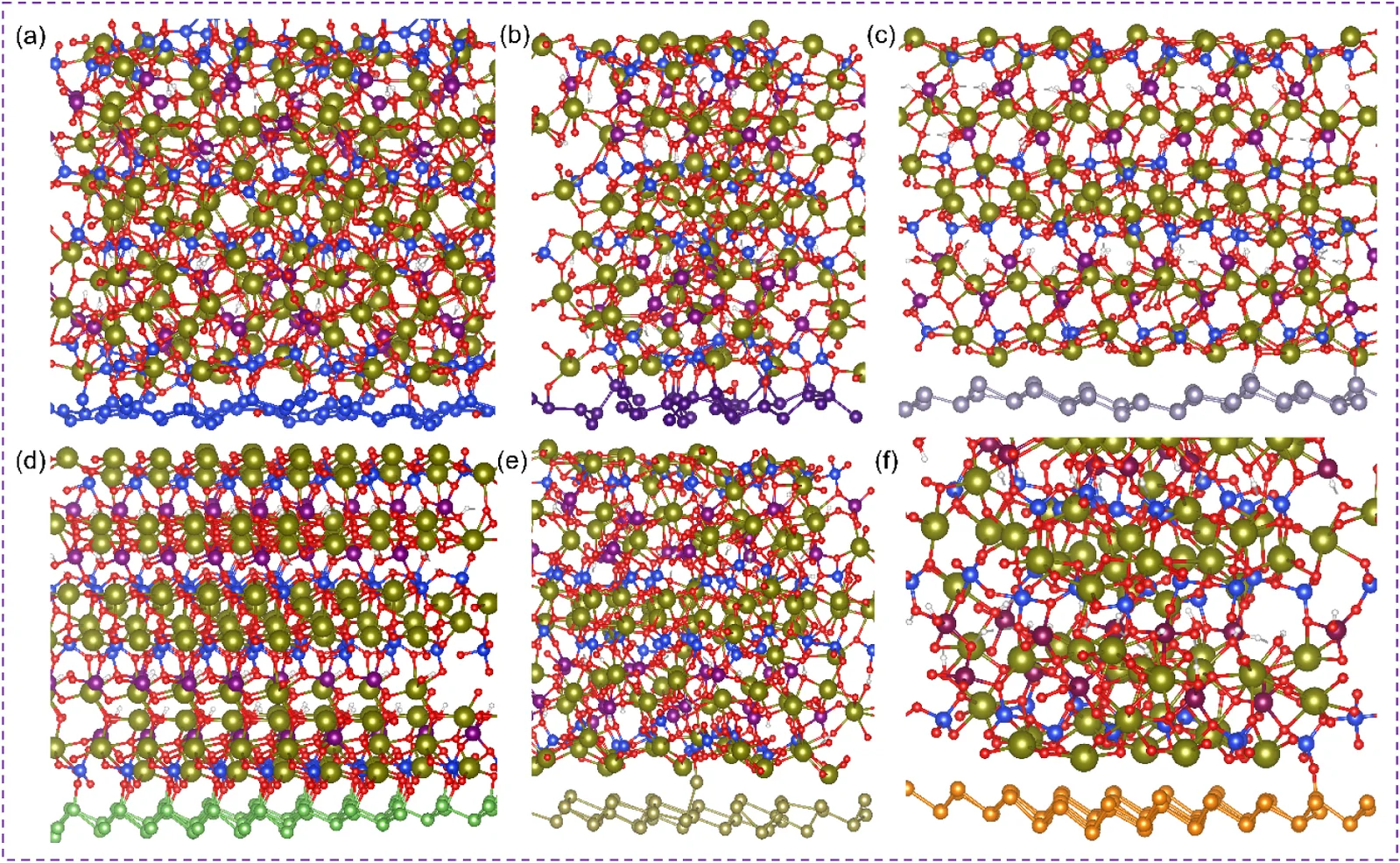
DFT analysis of C-A-S-H interaction with Xene-based 2D materials: (a) Silicene, (b) Germanene, (c) Stanene, (d) Arsenene, (e) Tellurene, and (f) Blue Phosphorus. Silicene (−56.26 eV) and germanene (−41.01 eV) exhibit strong covalent bonding with the C-A-S-H matrix, while arsenene (−20.73 eV) and tellurene (−14.95 eV) show moderate interactions. Stanene (−11.44 eV) and blue phosphorus (−9.43 eV) display weaker interfacial interactions dominated by limited bonding and charge transfer. [Calcium: gold, Oxygen: red, Silicon: blue, Aluminum: purple, Germanium: violet, Tin: silver, Arsenic: light green, Tellurium: olive-brown, Phosphorus: orange, Hydrogen: white].

## 4 Comprehensive Interfacial Chemistry of 2D Materials with C-A-S-H

A comprehensive DFT investigation was carried out to elucidate the interfacial interactions between C-A-S-H and a wide range of 2D materials, including Silicene, Germanene, Stanene, Arsenene, Tellurene, Blue Phosphorus, MoS_2_, WS_2_, Silicon Carbide, Boron Phosphate, Boron Nitride, and CrO_2_, along with functionalized systems such as MXene and graphene derivatives, as shown in Figure 8 and Table 1. The interfacial chemistry across these systems is fundamentally governed by the interplay of surface reactivity, orbital hybridization, and charge distribution, which collectively determine both the magnitude and nature of the interaction with the C-A-S-H matrix. In particular, Xene-based materials show strong chemical interactions, with Silicene and Germanene forming Si–O–Si and Si–O–Ge bonds with the silicate network, resulting in high interaction energies of −56.26 eV and −41.01 eV. The interaction energies, normalized by the surface area (eV/Å^2^), are also found to be large for these materials (Table 1). Arsenene, Tellurene, Stanene, and Blue Phosphorus exhibit weaker interactions (−20.73 to −9.43 eV) due to increased electron localization and reduced orbital overlap, which limit charge transfer at the interface.

**Table 1:** Summary of the Interaction Characteristics of the Studied 2D Materials with C-A-S-H

S. No.	2D Materials	Interactions	Interaction Energy (eV)^a^	Interaction Energy per unit area (eV/Å^2^)^a^
1.	Silicene	Covalent + electrostatic	–56.26	–0.21
2.	Germanene	Covalent + electrostatic	–41.01	–0.16
3.	Stanene	Electrostatic + vdW	–11.44	–0.02
4.	Arsenene	Electrostatic + Surface Adsorption	–20.73	–0.09
5.	Tellurene	VdW + Electrostatic	–14.95	–0.04
6.	Blue Phosphorus	Charge transfer	–9.43	–0.04
7.	Ti_3_C_2_T_*x*_	Proton exchange	-^a^	-^a^
8.	GO	Hydrogen bonding and electrostatic	–16.68	–0.06
9.	GO-SO_3_H	Strong ionic interaction	–0.64	0.00
10.	G-SH	Covalent and weak coordination	–11.09	–0.04
11.	GO-PO_3_H	Proton exchange	-^a^	-^a^
12.	MoS_2_	VdW	–2.01	–0.07
13.	WS_2_	VdW and surface adsorption	2.88	0.10
14.	V_2_C	Covalent	–44.09	–0.71
15.	Graphdiyne	Cation−π interaction	–4.35	–0.03
16.	SiC	Covalent bonding	–12.44	–0.33
17.	h-BN	VdW	–1.45	–0.03
18.	Boron phosphate	Ionic	–31.65	–0.28
19.	CrO_2_	Redox and electronic	–9.03	–0.07

a Proton transfer and structural reorganization occur when Ti_3_C_2_T_*x*_ and G-PO_3_H surfaces interact with C-A-S-H, rendering the calculation of interaction energies impractical. VdW = van der Waals interactions.

**Figure 8:**
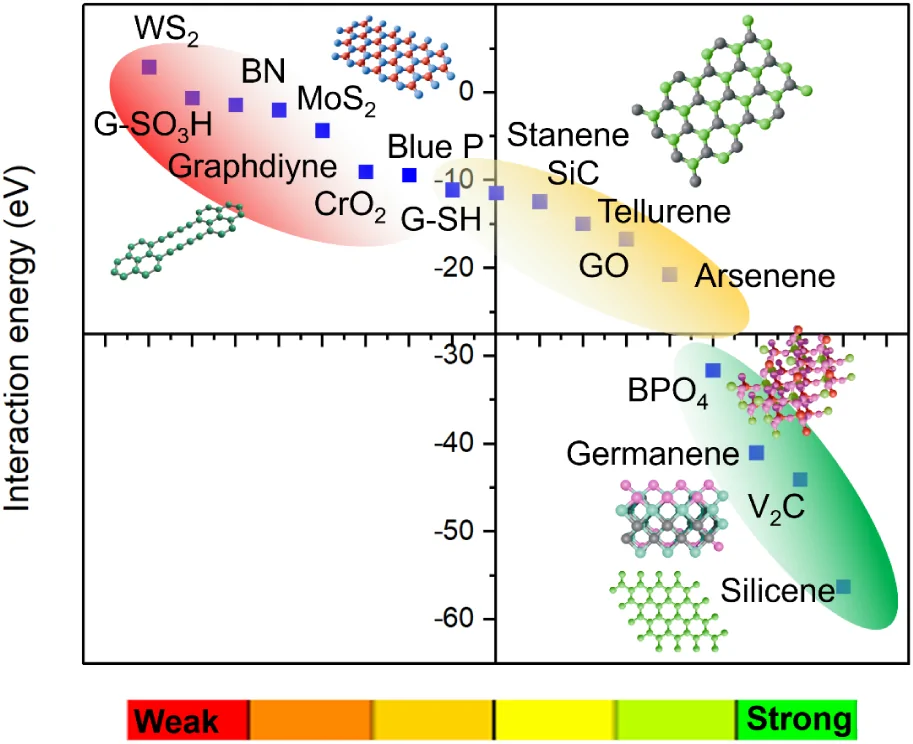
DFT-calculated interaction energies of 2D materials with C-A-S-H, showing interaction strength from weak (red) to strong (green). Xenes exhibit strong covalent bonding, while TMDs and oxides show weaker, coordination-driven interactions; functionalized MXenes and graphene enhance binding via hydrogen bonding and ionic effects.

TMDs, including MoS_2_ and WS_2_, interact with the C-A-S-H matrix primarily through Ca–S coordination and interfacial charge redistribution. In these systems, surface S atoms donate electron density to Ca^2+^ ions, leading to coordination-driven binding. The interaction energy of −2.01 eV for MoS_2_ indicates moderate interfacial coupling, whereas the positive value of +2.88 eV for WS_2_ reflects comparatively weaker and less favorable interaction, consistent with reduced orbital hybridization and limited charge transfer. Furthermore, carbide- and oxide-based systems show varied interfacial chemistries: boron phosphate is the most stable (−31.65 eV) via Ca–O and B–O–P linkages, silicon carbide forms Si–O–Si bonds (−12.44 eV), CrO_2_ interacts moderately through surface oxygen with Ca^2+^ (−9.03 eV), and BN remains largely inert due to a stable B–N bond. Functionalized MXenes facilitate proton exchange with the C-A-S-H network, enabling strong hydrogen bonding and ionic interactions. This promotes robust interfacial adhesion, improved dispersion, and enhanced mechanical integration within the geopolymer matrix. Similarly, graphene oxide and its derivatives, including sulfonated graphene oxide, thiographene, and phosphate graphene, provide a high density of functional groups that enable hydrogen bonding, ionic coordination, and surface complexation. These interactions enhance dispersion and interfacial compatibility while allowing flexibility and adaptive bonding within the matrix. Furthermore, the stability of the C-A-S-H composites was assessed via *ab initio* molecular dynamics simulations. Interactions between the C-A-S-H and the 2D materials were characterized using radial distribution function (RDF) analysis. As shown in Figure S10, the g­(*r*) curves exhibit prominent peaks below 2 Å, indicating strong covalent interactions persisting throughout the simulation. Overall, the wide variation in interaction energies, from highly negative values in covalently bonded systems to weak or even unfavorable interactions in coordination-dominated materials, demonstrates that interfacial stability is dictated not simply by dimensionality but by the underlying electronic structure and surface functionality.

Materials capable of forming direct covalent bonds or multisite ionic interactions achieve strong interfacial integration, whereas systems governed by weaker electrostatic or van der Waals forces contribute primarily through surface-level interactions. From the perspective of geopolymer concrete, these findings establish a direct link between nanoscale interfacial chemistry and macroscopic performance. Strongly interacting materials such as silicene, boron phosphate, and silicon carbide are expected to enhance matrix densification, mechanical strength, and durability through robust chemical integration. In contrast, MXenes and functionalized graphene systems offer chemically tunable interfaces that improve dispersion, ion transport, and multifunctional behavior. Materials with weaker interactions, such as CrO_2_ or WS_2_, may serve as functional additives, imparting properties such as electrical conductivity, lubrication, and environmental resistance without significantly altering the structural framework.

## 5 Conclusions

This study explores the potential of 2D materials as nanoscale reinforcements for sustainable geopolymer concrete, with a focus on improving mechanical performance, durability, and interfacial stability. Geopolymer systems, particularly those based on C-A-S-H, offer a low-carbon alternative to conventional Portland cement; however, their performance can be significantly enhanced through the incorporation of advanced nanomaterials. A comprehensive DFT investigation is performed on a wide class of materials, including Xenes, transition metal dichalcogenides, MXenes, graphene derivatives, and ceramic/covalent 2D systems such as silicon carbide, boron phosphate, boron nitride, and CrO_2_. The interfacial interactions between these materials and the C-A-S-H matrix are systematically analyzed in terms of adsorption energy, charge transfer, and bonding mechanisms. The study reveals that interfacial behavior is governed by a combination of covalent bonding, ionic coordination, hydrogen bonding, electrostatic interactions, and van der Waals forces. The results demonstrate that materials capable of forming covalent or ion-bridged interactions exhibit significantly stronger interfacial adhesion, leading to enhanced load transfer and matrix densification. In contrast, materials dominated by weak van der Waals interactions show limited reinforcement capability. Among the investigated systems, variations in electronic structure, surface chemistry, and functional groups play a critical role in determining interfacial stability and interaction strength. Overall, this work establishes a clear structure–property relationship between 2D material chemistry and C-A-S-H performance, providing fundamental insights into interfacial engineering. The findings offer a rational design strategy for developing next-generation geopolymer composites with improved mechanical strength, durability, and sustainability, while supporting the broader transition toward eco-friendly construction materials. While this study provides computational insights into 2D material–C-A-S-H interactions, future work will focus on integrating machine learning, experimental validation, and scalable synthesis strategies to bridge the gap between laboratory studies and industrial implementation, addressing the practical challenges of high-performance, sustainable construction materials.

## Supplementary Material



## Data Availability

The data underlying this study are available in the published article and its Supporting Information. Additional data are available in the Zenodo repository under the same title.

## Notes

Additional results from DFT calculations, including charge density difference plots, electronic structure analyses, and ab initio molecular dynamics (AIMD) simulations demonstrating structural stability (PDF)
