# Supplementary material for: Ab Initio Insights into the Interfacial Interactions of Emerging Two-Dimensional Nanomaterials with C‑A-S‑H Geopolymer Matrices
Source: ACS Omega. 2026 Oct 6;11(41):62906–18. doi: 10.1021/acsomega.6c04834 (PMC13688560; doi:10.1021/acsomega.6c04834)
Supplement: Supplementary file 1 [file ao6c04834_si_001.pdf]

Ab Initio Insights into the Interfacial Interactions of Emerging Two-Dimensional Nanomaterials with C-A-S-H Geopolymer Matrices

Rabindranath Lo,[1*] Anurag Misra,[2] and Kolleboyina Jayaramulu[3*]

[1]Institute of Organic Chemistry and Biochemistry, Czech Academy of Sciences, v.v.i., Flemingovo nám. 2, 16000 Prague 6, Czech Republic.

[2]Department of Civil Engineering, Indian Institute of Technology Jammu, Jammu & Kashmir, 181221, India.

[3]Hybrid Porous Materials Laboratory, Department of Chemistry, Indian Institute of Technology Jammu, Jammu & Kashmir, 181221, India.

Key words: Geopolymers; Calcium Aluminosilicate Hydrate (C-A-S-H); Density Functional Theory (DFT); Two-Dimensional Materials; Adsorption Energy; Charge Transfer.

Email (s): rabindranath.lo@uochb.cas.cz; jayaramulu.kolleboyina@iitjammu.ac.in

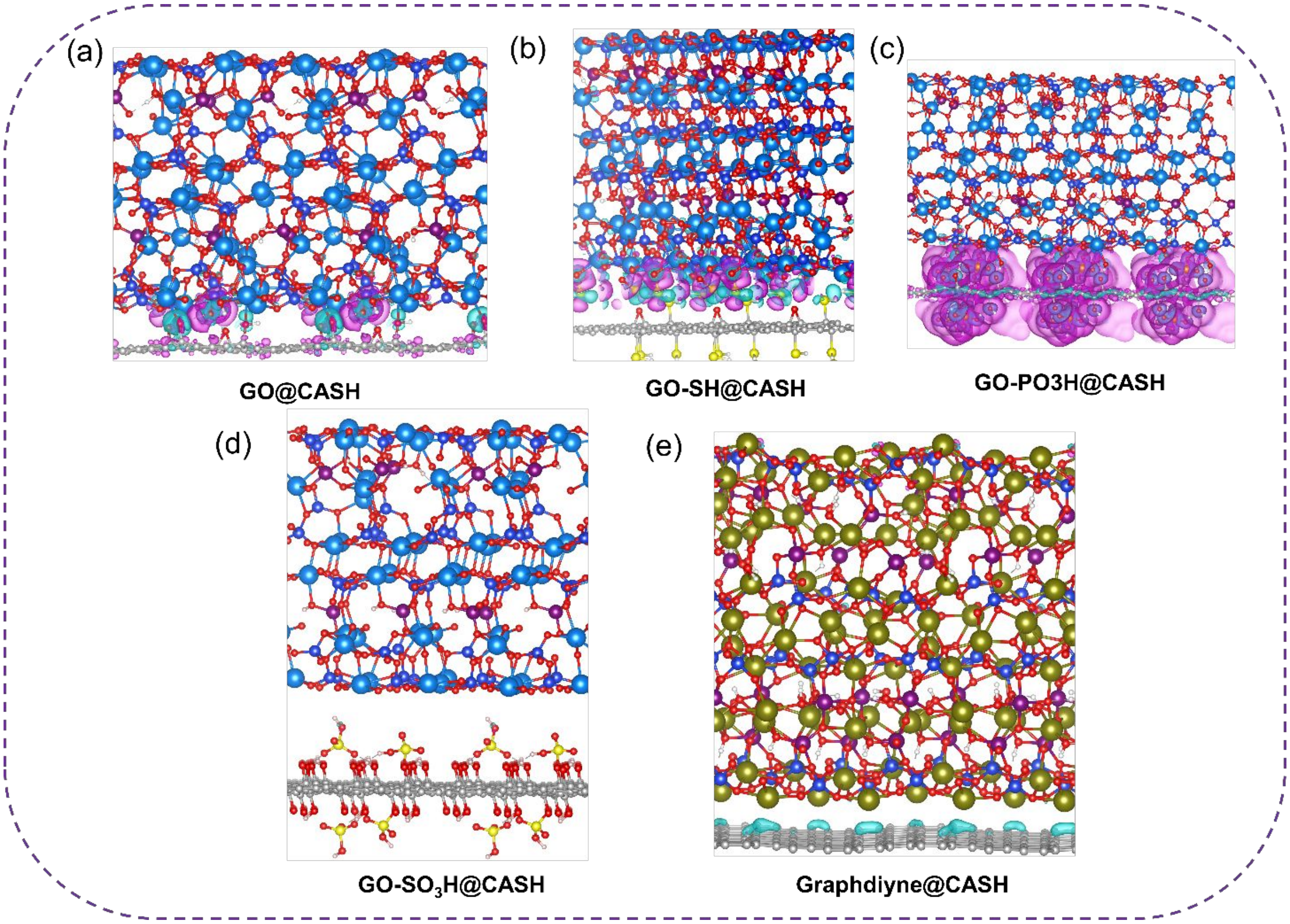

Figure S1 Calculated differential charge density of graphene derivatives on C-A-S-H (a) GO (b) G-SH (c) G-PO_3H (d) G-SO_3H and (e) Graphdiyne. Pink and cyan isosurfaces represent charge accumulation and depletion, respectively. [Calcium: gold, Oxygen: red, Silicon: blue, Aluminium: purple, Carbon: grey, Hydrogen: white, Phosphorus: orange, Sulfur: yellow]

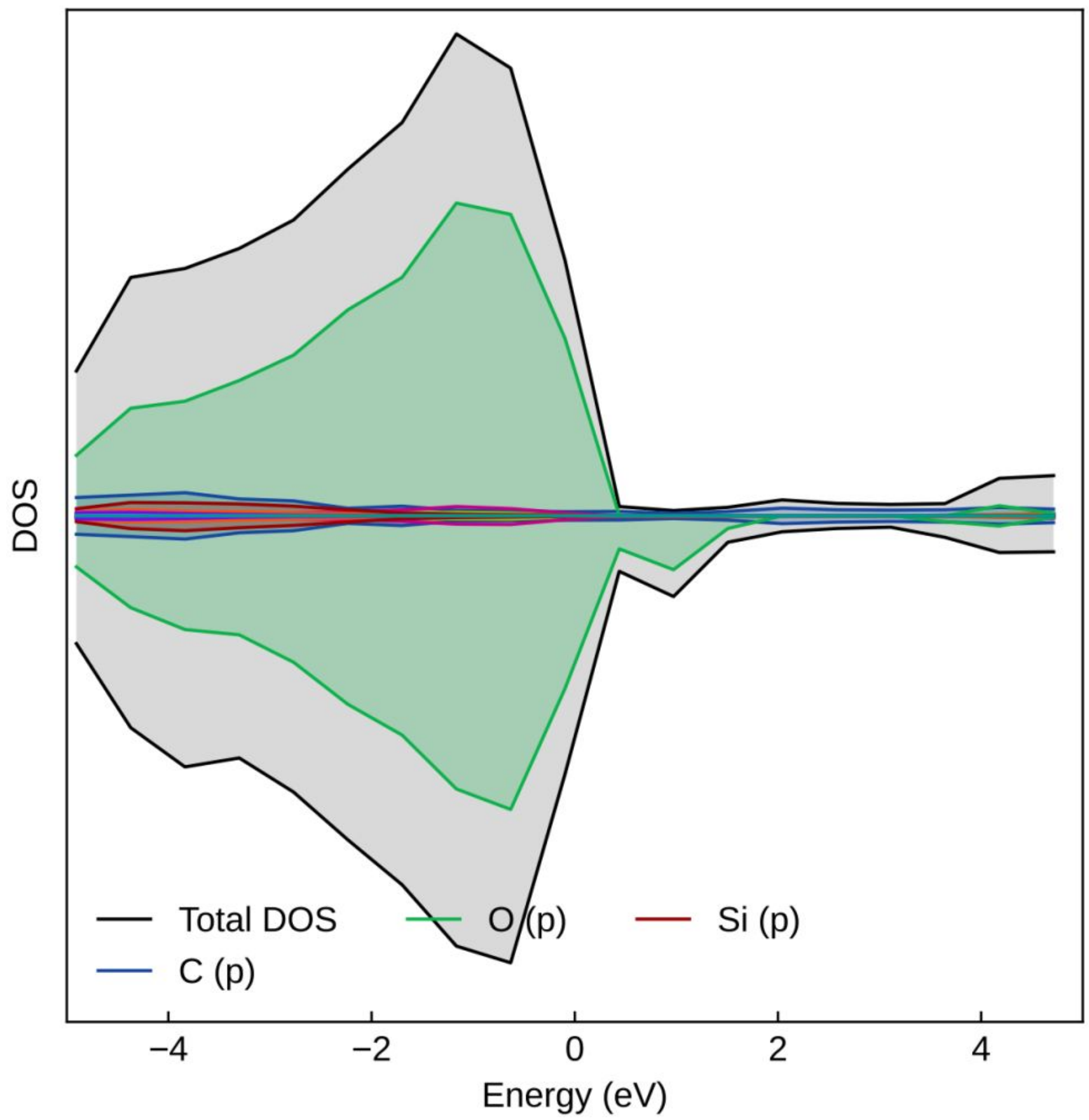

Figure S2 Total and partial electronic density of states (TDOS and PDOS, respectively) of C-A-S-H interaction with Graphdiyne.

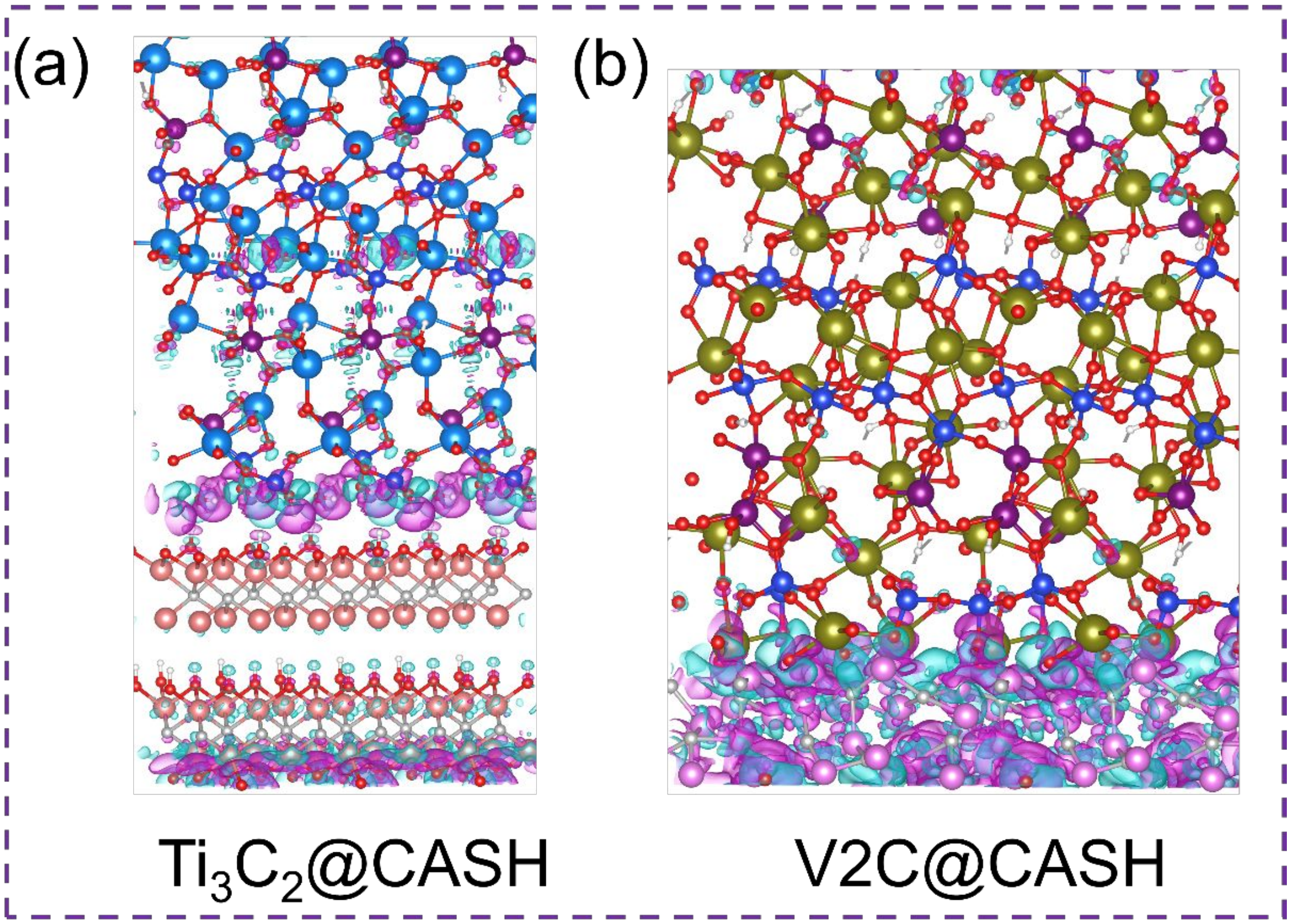

Figure S3 Calculated differential charge density of (a) Ti_3C_2Tx (-OH, -F) and (b) V_2C MXenes on the C-A-S-H surface. Pink and cyan isosurfaces represent charge accumulation and depletion, respectively. [Calcium: gold, Oxygen: red, Silicon: blue, Aluminium: purple, Titanium: pink, Vanadium: bright pink, Carbon: grey, Hydrogen: white]

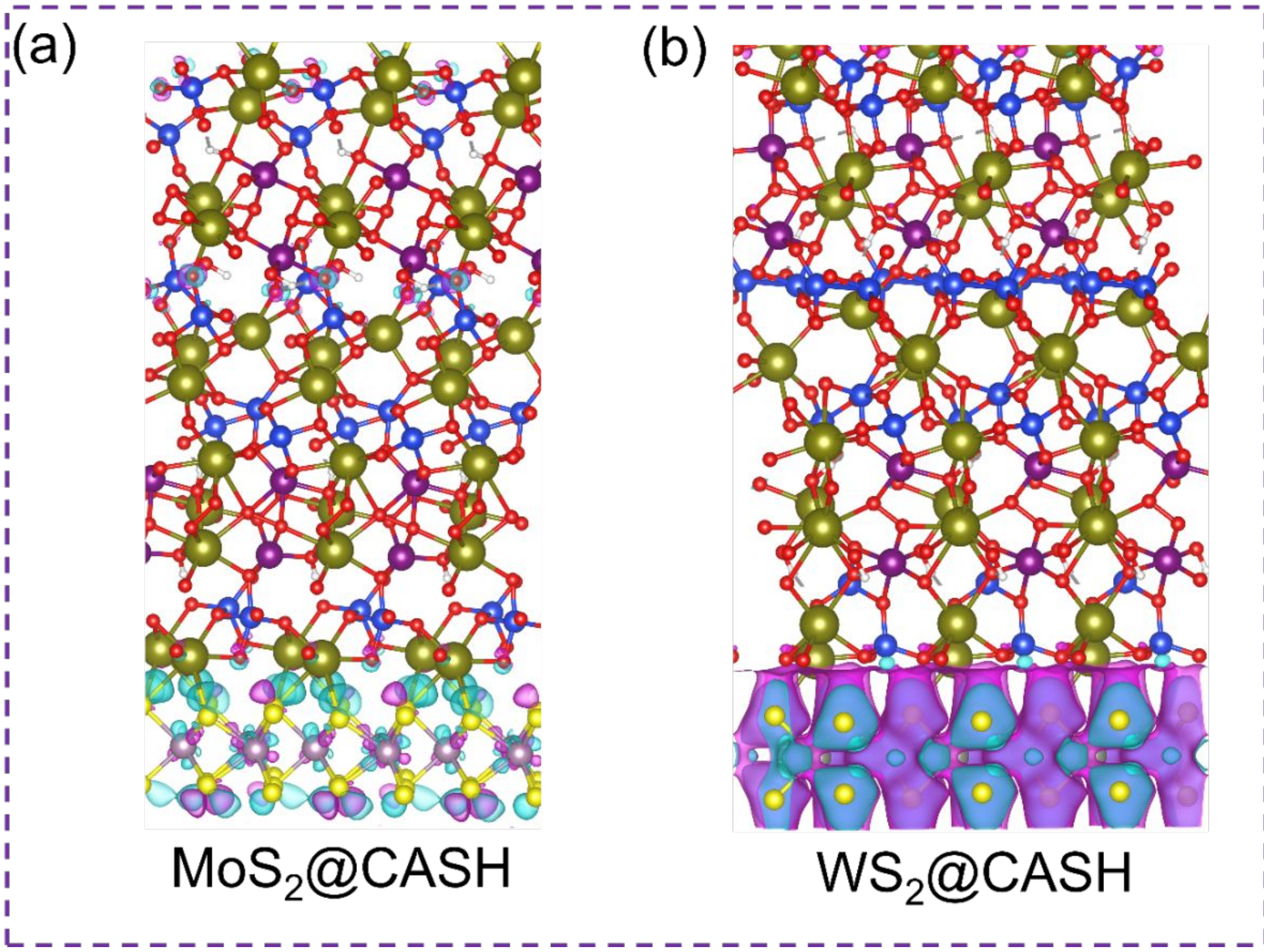

Figure S4 Calculated differential charge density of C-A-S-H interaction with (a) MoS_2 and (b) WS_2. Pink and cyan isosurfaces represent charge accumulation and depletion, respectively. [Calcium: gold, Oxygen: red, Silicon: blue, Aluminium: purple, Sulfur: bright yellow, Molybdenum: lavender, Tungsten: dark grey, Hydrogen: white]

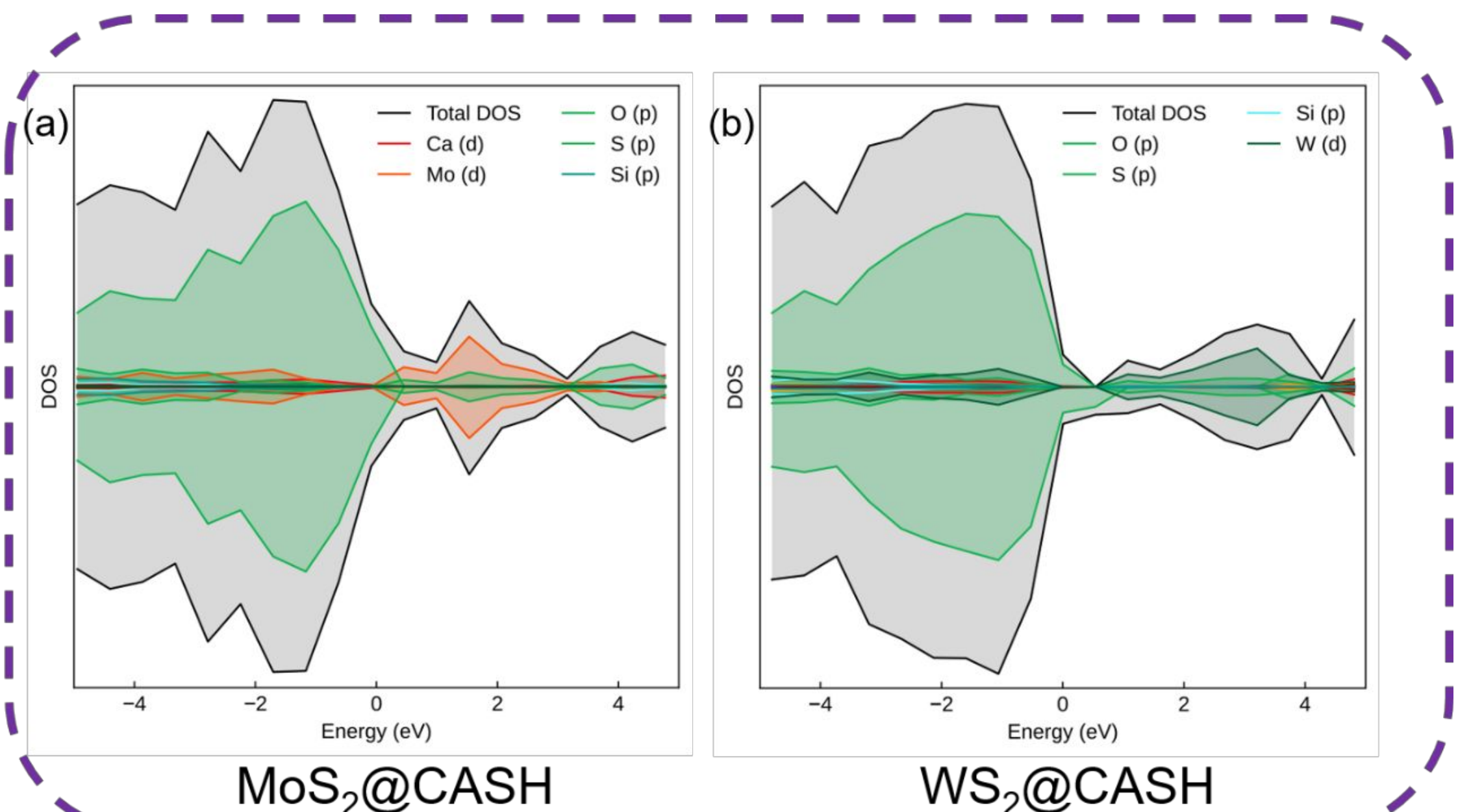

Figure S5 Total and partial electronic density of states (TDOS and PDOS, respectively) of C-A-S-H interaction with (a) MoS_2 and (b) WS_2.

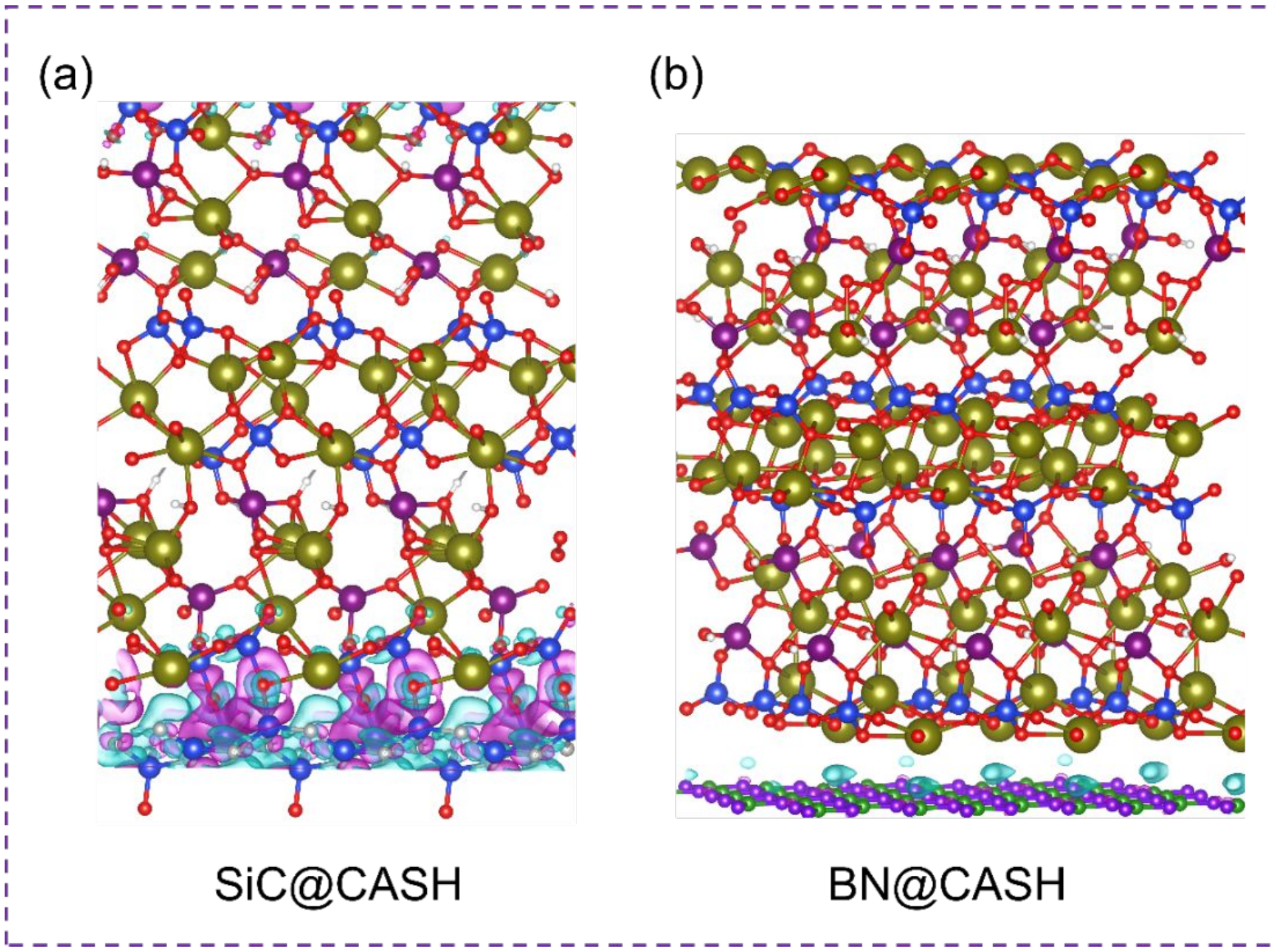

Figure S6 Calculated differential charge density of C-A-S-H interaction with (a) Silicon Carbide and (b) h-BN. Pink and cyan isosurfaces represent charge accumulation and depletion, respectively. [Calcium: gold, Oxygen: red, Silicon: blue, Aluminium: purple, Carbon: light grey, Boron: green, Nitrogen: violet, Hydrogen: white]

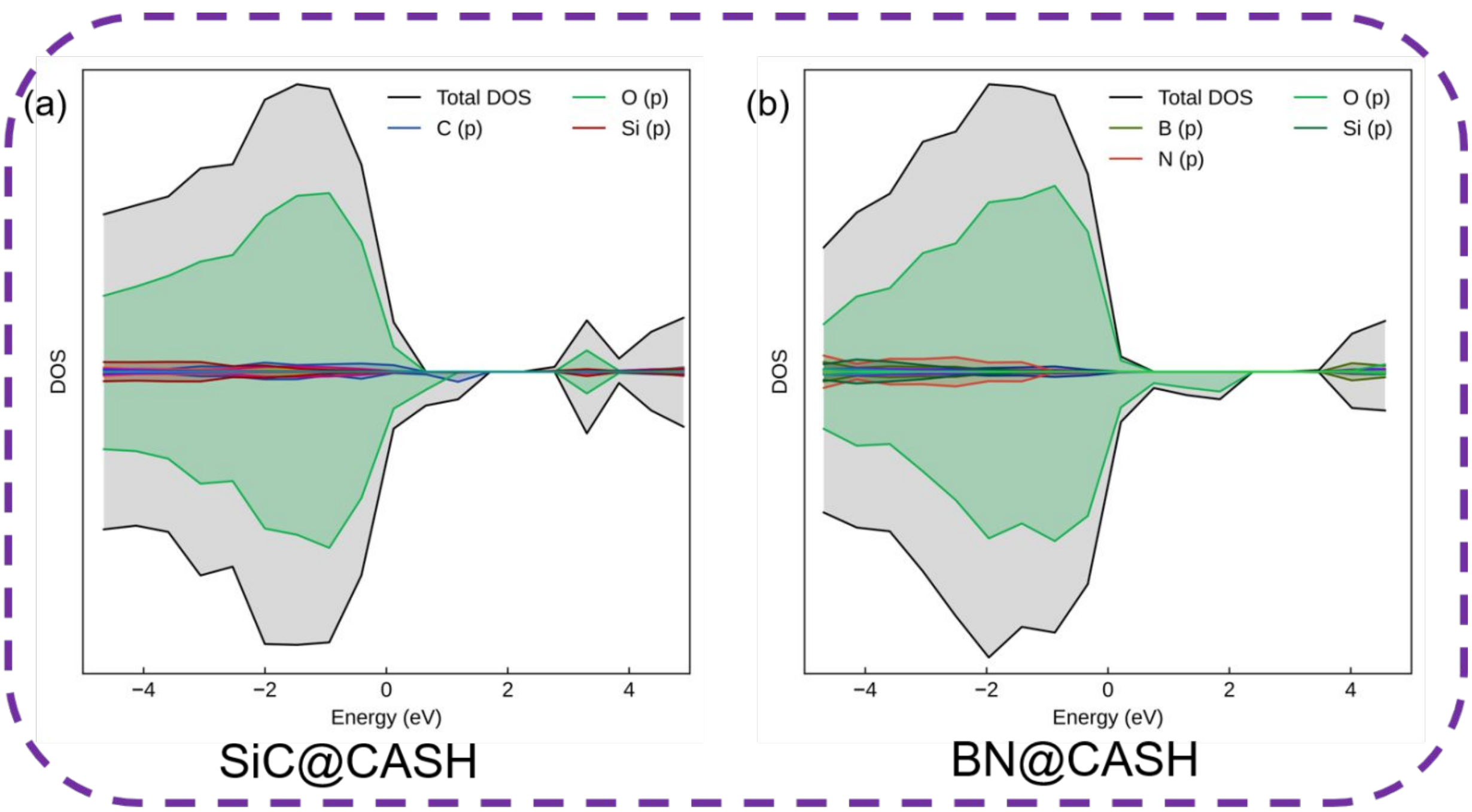

Figure S7 Total and partial electronic density of states (TDOS and PDOS, respectively) of C-A-S-H interaction with (a) Silicon Carbide and (b) h-BN.

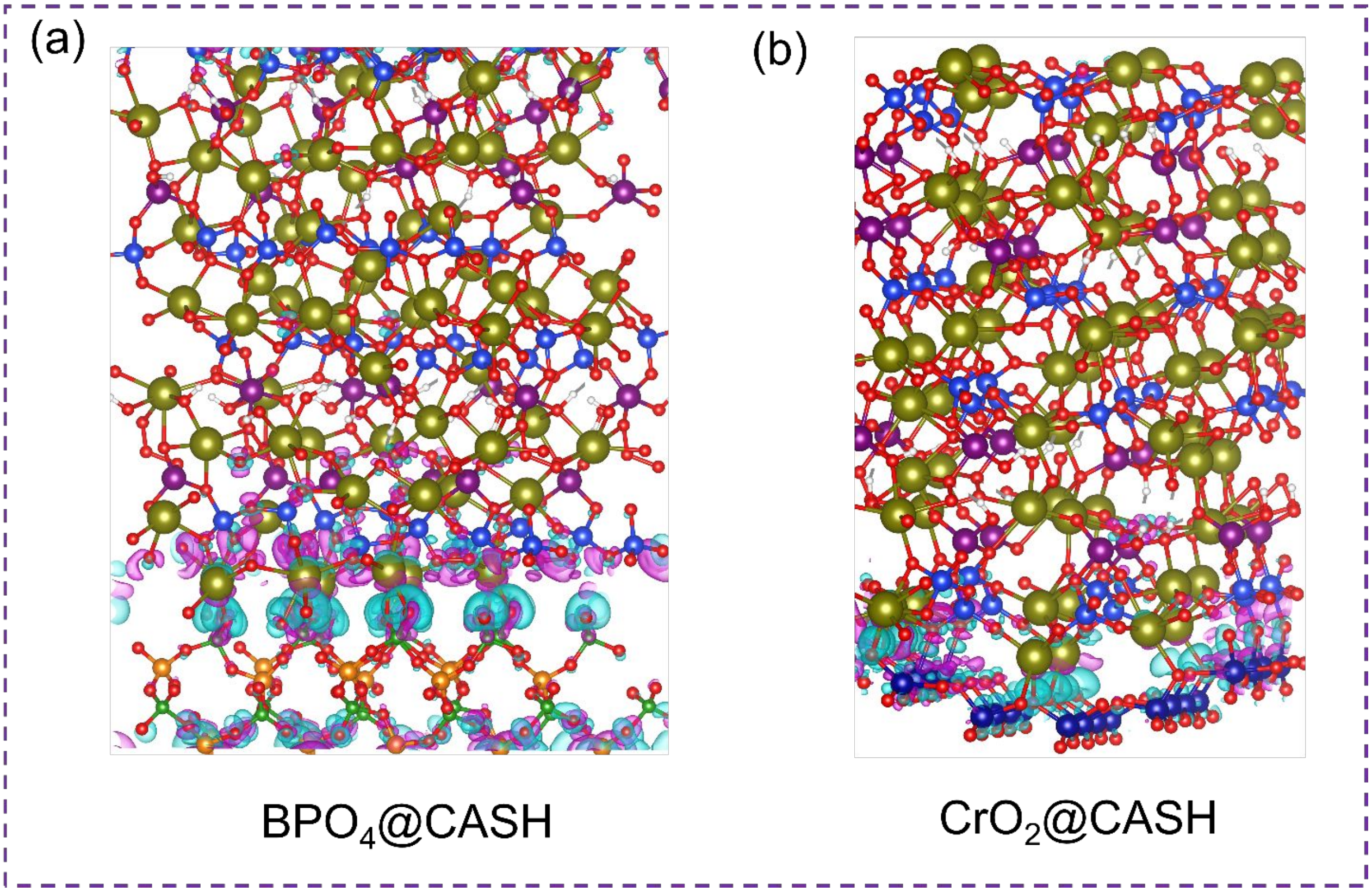

Figure S8 Calculated differential charge density of C-A-S-H interaction with (a) Boron phosphate and (b) CrO_2. Pink and cyan isosurfaces represent charge accumulation and depletion, respectively. [Calcium: gold, Oxygen: red, Silicon: blue, Aluminium: purple, Boron: green, Chromium: dark purple, Phosphorus: orange, Hydrogen: white]

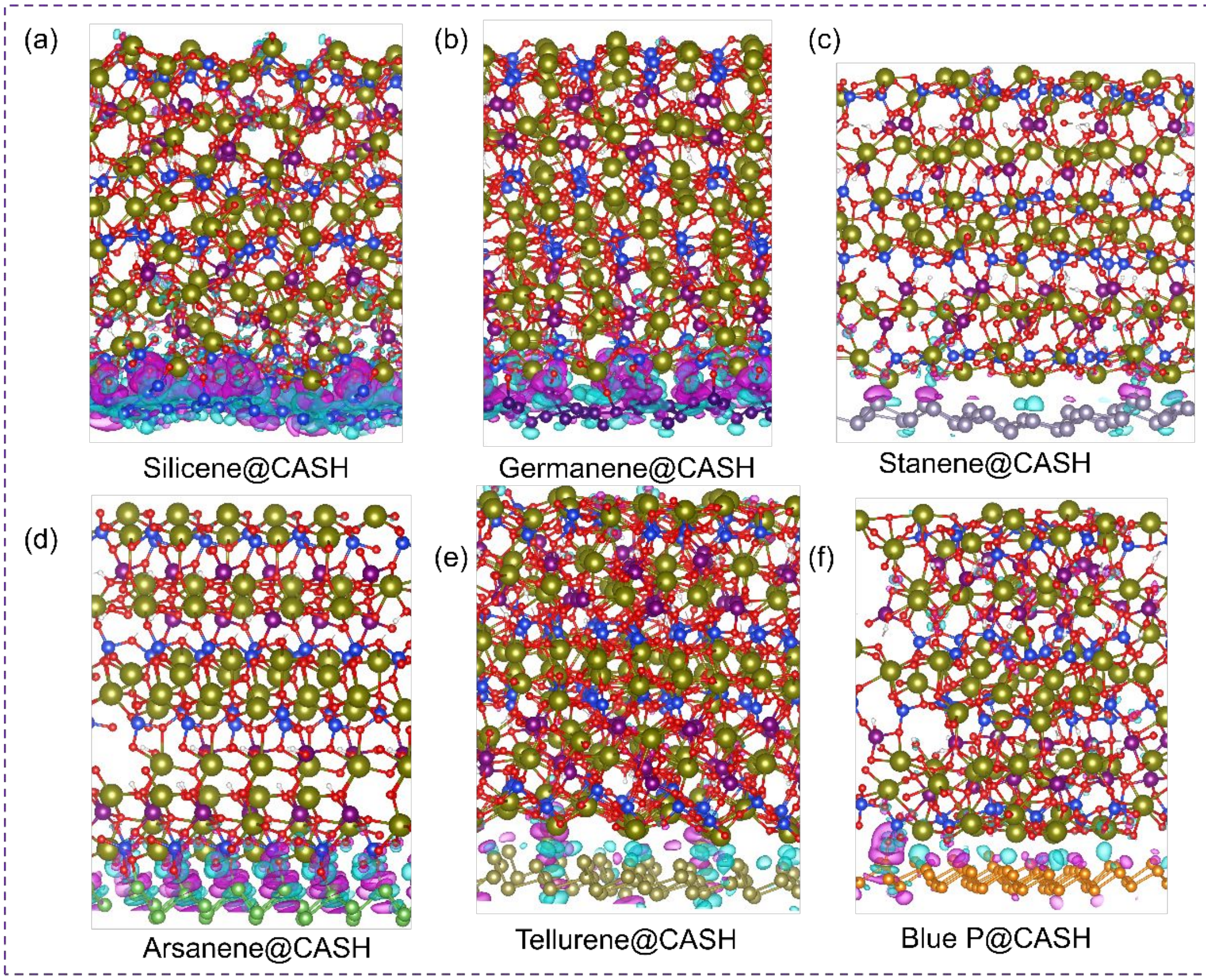

Figure S9 Calculated differential charge density of C-A-S-H interaction with Xene-based 2D materials: (a) Silicene, (b) Germanene, (c) Stanene, (d) Arsenene, (e) Tellurene, and (f) Blue Phosphorus. Pink and cyan isosurfaces represent charge accumulation and depletion, respectively.[Calcium: gold, Oxygen: red, Silicon: blue, Aluminium: purple, Germanium: violet, Tin: silver, Arsenic: light green, Tellurium: olive-brown, Phosphorus: orange, Hydrogen: white]

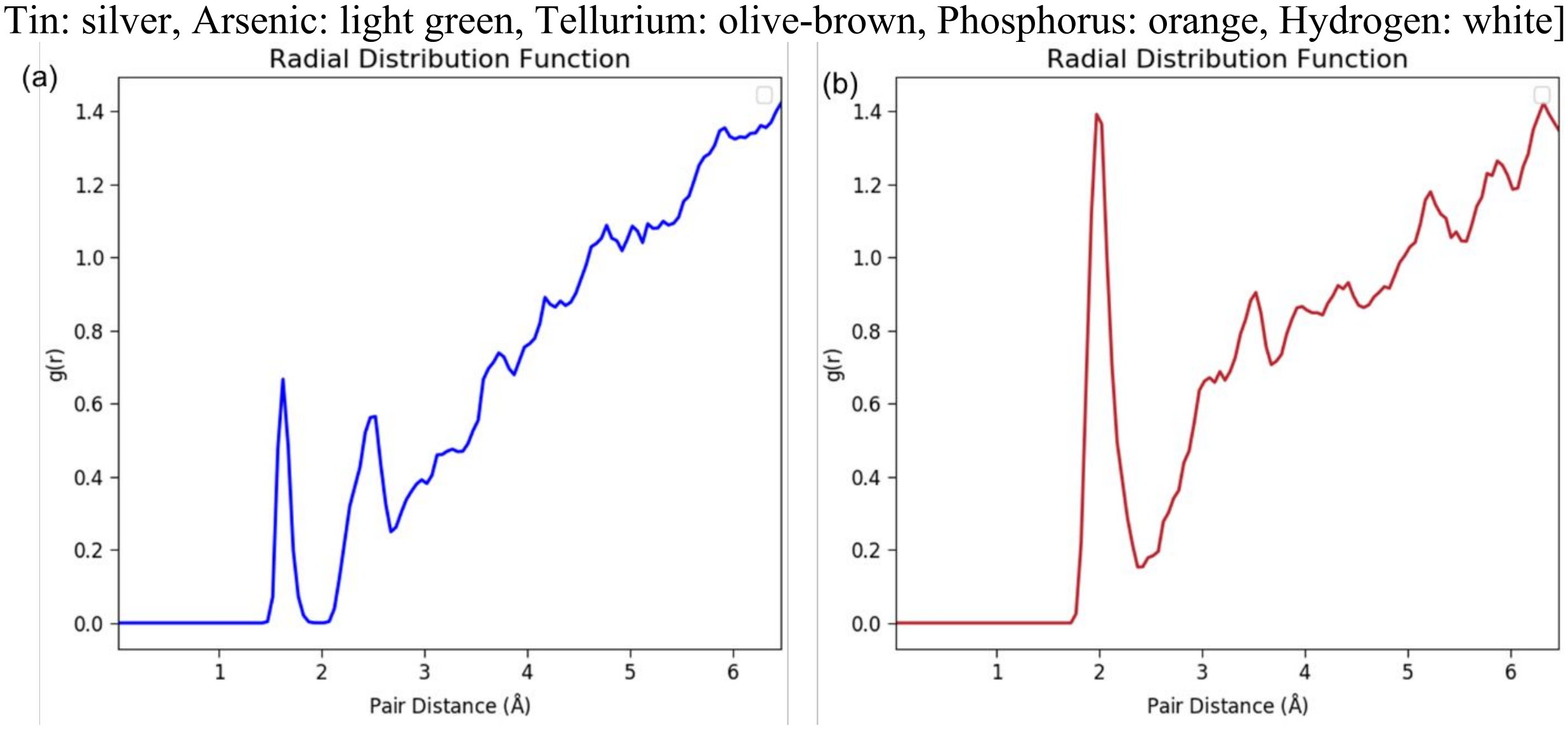

Figure S10 The radial distribution functions, g(r), are shown for the geopolymer composites with a) Boron phosphate and b) Vanadium Carbide.

Table S1 Cell volumes (a × b × c) used in geometry optimization for each investigated hybrid systems. Values are given in Ångströms and rounded to two decimal places.

**2-D Materials**	a	b	c
Silicene	16.19	16.19	35.00
Germanene	15.95	15.95	35.00
Stanene	18.68	32.35	35.00
Arsenene	19.55	11.29	30.00
Tellurene	27.16	13.34	32.00
Blue Phosphorus	16.39	16.34	52.65
MoS_2	5.47	5.47	32.00
WS_2	5.46	5.46	32.00
Vanadium Carbide	5.74	10.79	32.00
Graphdiyne	10.16	13.58	30.00
Silicon carbide (SiC)	6.17	6.17	30.00
Boron nitride (h-BN)	6.63	6.63	30.00
Boron phosphate	18.31	6.28	35.00
CrO_2	22.11	6.25	35.00
